# Potential Neural Mediators of Mom Power Parenting Intervention Effects on Maternal Intersubjectivity and Stress Resilience

**DOI:** 10.3389/fpsyt.2020.568824

**Published:** 2020-12-08

**Authors:** S. Shaun Ho, Maria Muzik, Katherine L. Rosenblum, Diana Morelen, Yoshio Nakamura, James E. Swain

**Affiliations:** ^1^Department of Psychiatry and Behavioral Health, Renaissance School of Medicine at Stony Brook University, Stony Brook, NY, United States; ^2^Departments of Psychiatry, Obstetrics & Gynecology, University of Michigan, Ann Arbor, MI, United States; ^3^Department of Psychology, East Tennessee State University, Johnson City, TN, United States; ^4^Department of Anesthesiology, Pain Research Center, University of Utah School of Medicine, Salt Lake City, UT, United States

**Keywords:** intersubjectivity, empathy, parenting intervention, parenting stress, amygdala, dorsomedial prefrontal cortex, PAG = periaqueductal gray, Bayesian active inference

## Abstract

Stress resilience in parenting depends on the parent's capacity to understand subjective experiences in self and child, namely intersubjectivity, which is intimately related to mimicking other's affective expressions (i. e., mirroring). Stress can worsen parenting by potentiating problems that can impair intersubjectivity, e.g., problems of “over-mentalizing” (misattribution of the child's behaviors) and “under-coupling” (inadequate child-oriented mirroring). Previously we have developed Mom Power (MP) parenting intervention to promote maternal intersubjectivity and reduce parenting stress. This study aimed to elucidate neural mechanisms underlying the effects of MP with a novel Child Face Mirroring Task (CFMT) in functional magnetic-resonance-imaging settings. In CFMT, the participants responded to own and other's child's facial pictures in three task conditions: (1) empathic mirroring (Join), (2) non-mirroring observing (Observe), and (3) voluntary responding (React). In each condition, each child's neutral, ambiguous, distressed, and joyful expressions were repeatedly displayed. We examined the CFMT-related neural responses in a sample of healthy mothers (*n* = 45) in Study 1, and MP effects on CFMT with a pre-intervention (T1) and post-intervention (T2) design in two groups, MP (*n* = 19) and Control (*n* = 17), in Study 2. We found that, from T1 to T2, MP (vs. Control) decreased parenting stress, decreased dorsomedial prefrontal cortex (dmPFC) during own-child-specific voluntary responding (React to Own vs. Other's Child), and increased activity in the frontoparietal cortices, midbrain, nucleus accumbens, and amygdala during own-child-specific empathic mirroring (Join vs. Observe of Own vs. Other's Child). We identified that MP effects on parenting stress were potentially mediated by T1-to-T2 changes in: (1) the left superior-temporal-gyrus differential responses in the contrast of Join vs. Observe of own (vs. other's) child, (2) the dmPFC-PAG (periaqueductal gray) differential functional connectivity in the same contrast, and (3) the left amygdala differential responses in the contrast of Join vs. Observe of own (vs. other's) child's joyful vs. distressed expressions. We discussed these results in support of the notion that MP reduces parenting stress via changing neural activities related to the problems of “over-mentalizing” and “under-coupling.” Additionally, we discussed theoretical relationships between parenting stress and intersubjectivity in a novel dyadic active inference framework in a two-agent system to guide future research.

## Introduction

Parent-child interactions are crucial for child development and sources of joyful or distressed experiences in the dyad. However, when stress compromises a parent's parenting capacity, parent-child interactions tend to deteriorate and exacerbate parental stress in return (1, 2). Parental intersubjectivity, described below, has been identified as a key resilience factor, and a target of parenting interventions, to buffer the adverse effects of parental stress or depressive moods on parent-child interactions (3, 4).

Intersubjectivity is defined here as the understanding of self's and other's internal, covert states (e.g., internal models, intention, and feeling). Parental intersubjectivity enables a parent to feel what a child's subjective experience or mind *is like*, while maintaining the distinctive awareness of self and child's subjective experiences (first-person and second-person subjectivity). Synonymous to interpersonal understanding (5) and some, but not all, definitions of empathy (6), parental intersubjectivity lies in the core of several parenting-related constructs, such as parental empathic attunement (7), parental reflective functioning (8, 9), parental sensitivity (10, 11), and parental embodied mentalizing (12). All these complex constructs point to a parent's capacity to utilize dyadic interactions to achieve valid attributions of the child's covert states underlying overt behaviors. Thus, in lieu of other terms, the term *intersubjectivity* is used here to emphasize its reliance on person-person interactions (the prefix, *inter*) and its focus on the awareness of self and other's lived experiences (*subjectivity*).

A key attribute underlying intersubjectivity is spontaneous mimicry or voluntary imitation of others' facial expressions or manual gestures. Infants show spontaneous facial mimicry soon after birth (13), which fits the onset of the development of intersubjectivity (14). Mothers with secure parent-child bonding show greater child-oriented face mirroring (15). Notably, mirroring can be performed spontaneously without activating higher-order representations (16). The dissociation between mirroring and higher-order representations points to dissociable processes or systems that may underlie mirroring others' actual behaviors vs. mentally representing (or thinking about) others.

Development of *intersubjectivity* begins in infancy (14), and remains plastic throughout the lifespan, for better or worse, bearing prominent clinical and societal significance (4, 7, 17–20). Mothers exposed to interpersonal violence (21) or suffering depressive mood disorders (4) may show impairment in intersubjectivity, leaving them at risk for excessive parenting stress, as parenting stress is inversely associated with parental intersubjectivity (22).

In this paper, we present a translational neuroscience study to elucidate potential neural mediators of an intersubjectivity-promoting parenting intervention that aims to reduce maternal parenting stress. We address this topic at two levels of analysis, one at an *empirical* level (elaborated here) and the other at an *abstract* level (elaborated in section Abstract Level of Analysis—Toward an Overarching Framework for Research on Intersubjectivity). We begin with the description of two problems that may impair intersubjectivity, namely “over-mentalizing” and “under-coupling” problems, then discuss our parenting intervention, Mom Power (MP), that reverses these intersubjectivity problems. Next, we present brain systems underlying these intersubjectivity problems in two functional magnetic resonance imaging (fMRI) studies utilizing a novel fMRI task. We end with a brief theoretical discussion on the dyadic active inference framework (with extensive elaboration in section Abstract Level of Analysis—Toward an Overarching Framework for Research on Intersubjectivity) to link intersubjectivity with parenting stress, which in turn may theoretically account for the “over-mentalizing” and “under-coupling” problems that are commonly observed in clinical settings.

### Intersubjectivity Impaired by “Over-Mentalizing” and “Under-Coupling” Problems

Impaired parental intersubjectivity frequently manifests as a parent's rigid misattributions of a child's unwelcome behavior to malevolence. For example, a mom may think her son's defiance to her requests means ill to her, “*he did it to humiliate me*.” When repeated misattributions of the child consolidate into a rigid belief, the parent may interpret all difficulties in parenting as a character flaw in the child, “*he is mean*.” Such problem is called **“over-mentalizing,”** i.e., the parent overly mentalizes the child into a generalization without relying on situational cues into circular reasoning “*he defies me to humiliate me because he is mean*.” Holding on to such a misbelief, the parent can develop a judgmental stance toward the child, which subsequently predicts chronic rejection, rage toward the child, parent-child bonding problems, and parental depressive moods (23). Furthermore, when parents habitually over-mentalize the child, they ignore situational, emotional, and behavioral cues in the “real-time” parent-child interactions that could otherwise serve as bottom-up data to rectify the parents' misbeliefs (24). Such obliviousness is called **“under-coupling,”** i.e., the parent is disengaged from observing how their physical or verbal actions (e.g., negative judgments or rejections) make their child feel and may “induce” the observed behaviors. Both “over-mentalizing” and “under-coupling” are undesired mental state manifestations and indicators of impaired parental intersubjectivity. When parents experience heightened parental stress, their defensive reactions (e.g., fight or flight) become sensitized, and “over-mentalizing” and “under-coupling” phenomena can worsen, further exacerbating impairment of parental intersubjectivity in a vicious cycle.

### Mom Power—An Intersubjectivity-Promoting Parenting Intervention

To mitigate parenting problems and reduce parenting stress, our team has developed MP, a group parenting intervention that fosters maternal intersubjectivity in clinical settings. For details on the intervention delivery, please see elsewhere (25). Impact on intersubjectivity is thought to be accomplished through (1) interpersonal, interactive exchanges with group peers to facilitate implicit imitations and explicit empathy-boosting exercises, (2) hands-on acquisition of knowledge regarding child's developmental needs to rectify developmental expectations and improve mothers' working models/mental representations of their child, (3) non-judgmental mindfulness practice to support regulation of own distress, which in turn inhibits mothers' defensive reactions to stress, and (4) enhancement of reflective capacity to build the awareness of self and other's lived experiences and needs (24, 26–28). Previously, we have found that MP reduces parenting stress (27), corrects developmentally-inappropriate, distorted working models/mental representations of their child (28), and modulates maternal brain responses to baby cry stimuli as a function of parenting stress (29). Based on this work, we postulate that MP will reverse both maternal intersubjectivity problems, “over-mentalizing” and “under-coupling” (28), which in turn will reduce maternal parenting stress.

### Brain Systems Underlying Intersubjectivity

The social neuroscience literature suggests that the recognition and attribution of goals and intentions of another person's behaviors is primarily supported by three distinct but inter-related neural systems, namely ***mirroring system***, ***mentalizing system*, and** ***salience*** ***network***, described below (30–32). **The** ***mirroring system***becomes active when an agent performs an action or perceives another agent's similar action (33). The co-localization of activities related to perception and action in the brain affords an observer's automatic recognition of the immediate goal of the other agent's actions. This system involves the posterior inferior frontal gyrus (pIFG), dorsal and ventral premotor cortex (dPMC and vPMC), supplemental motor area (SMA), inferior parietal lobule (IPL), superior parietal lobule (SPL), intraparietal sulcus (IPS), superior temporal gyrus (STG), and pericentral cortex (34–39).

**The** ***mentalizing system***becomes active when a person is attributing mental states to others and this system involves the precuneus/posterior cingulate cortex (PrC/PCC), dorsal, middle, and ventral medial prefrontal cortices (dmPFC, mmPFC, and vmPFC, respectively), posterior temporal sulcus (pSTS), temporal pole, and temporoparietal junction (TPJ) (32). A meta-analysis suggests that the dmPFC, mmPFC, vmPFC, and PrC/PCC form a loop to generate narrative thoughts related to affective representations of self and other (40). In this loop, interpersonal scripts (autobiographical stories) are generated when the PrC/PCC, as a thought generator (41), connects affective potentials stored in the vmPFC (42) to regions that serve as a proximal-object sketchpad that represents the self (in mmPFC) (43, 44) or a distal-object sketchpad that represents another person (in dmPFC) (43). The dmPFC-dependent functional connectivity preferentially participates in mentalization in verbal, but not in visual, modality, while the TPJ-dependent functional connectivity participates in both modalities (45). Thus, the dmPFC represents others' enduring attributes (a generic image of other's identity) without differentiating self and other's perspectives (40, 46). In contrast, the TPJ represents other's inner thoughts that are different from one's own perspective, with self-other distinction (47) and mediates inferences about others, such as their transient goals, desires and beliefs (48). Moreover, the anterior part of TPJ is involved in joint attention, which requires spatial representation of other's attentional direction (30).

**The** ***salience network***includes dorsal ACC, posterior ventral MCC, bilateral anterior insula cortices (IC), and subcortical regions such as PAG, hypothalamus, thalamus, midbrain, striatum, and extended amygdala (49). This network detects internal and external events that are personally meaningful (50) and interacts with the *mentalizing system* to respond to attachment figures (51). Indeed, the salience network largely overlaps with a *maternal caregiving system* that regulates parenting behaviors, including the amygdala, IC, and two motivational sub-systems—one for affiliative motivations that include the hypothalamus, ventral tegmental area (VTA), nucleus accumbens (NAc), and ventral pallidum (VP) and the other for defensive (fight or flight) motivation mediated by the periaqueductal gray (PAG) (52–54). Notably, many of these regions (e.g., the amygdala, PAG, and NAc) are sensitive to *signed prediction errors* of reward or punishment with reference to preceding baselines, i.e., activated when detecting a greater-than-expected level of salience (positive prediction errors of reward or punishment, e.g., the presence of unexpected salience) and deactivated when detecting a less-than-expected level of salience (negative prediction errors of reward or punishment, e.g., the omission of expected salience). For examples, the NAc is sensitive to *signed prediction errors* of reward (55, 56); the amygdala is sensitive to *signed prediction errors* of reward (e.g., desirable liquid) and/or punishment (e.g., undesirable air-puff) (57); besides, the amygdala is also sensitive to the *signed prediction errors* in aversive stimuli, e.g., activated when detecting the presence of unexpected foot shock and deactivated when detecting the omission of expected foot shock (58). Notably, consistent with the notion that NAc and PAG served as opponent motivations of reward-seeking and defense (flight-or-flight) respectively, the NAc and PAG responded in opposite manners to aversive prediction errors, as unexpected pain not only deactivated the NAc (a negative prediction error of reward as if the unexpected pain was equivalent to the omission of reward), but also activated PAG (a positive prediction error of punishment as if the unexpected pain was equivalent to the presence of unexpected punishment) (59). As described later, the contrast of mirroring the child's joyful vs. distressed expressions in our experimental task is computed to index the sensitivity of *signed prediction errors* specific to maternal mirroring of own child's emotions.

The *mirroring system* largely overlaps with the frontoparietal network (60); the *mentalizing system* largely overlaps with the default-mode network that is more active during resting states (61) and mind wandering (62), as compared to states of actively paying attention to the environments. Spontaneous activities in the default-mode network are often anti-correlated with those in the frontoparietal network (63). Thus, we postulate that empathic mirroring of others encompasses bottom-up perception-action coupling between two agents, which can potentially activate the *mirroring system* and automatically deactivate the *mentalizing system*, as compared to (non-mirroring) observing others.

Moreover, as virtually all cognitive processes depend on the functional connectivity among participating brain networks (64), the functional connectivity among the three aforementioned brain systems, *i.e., mirroring system, mentalizing system, and salience network*, are key to intersubjectivity (45). Indeed, the capacity for intersubjectivity seems to depend on the functional connectivity between the dmPFC (in the *mentalizing system*) and the inferior frontal gyrus (in the *mirroring system*) (65). Notably, the functional connectivity between stress-dependent brain regions (which include the *salience network*) and the child-representing regions, i.e., dmPFC, may underlie the stress-potentiation of the “over-mentalizing” problem. It is through functional connectivity that the *salience network* may switch up or down the activity in the frontoparietal network and the default-mode network *alternately* (66). These results underscore the roles of dmPFC-dependent functional connectivity in representing the child in maternal intersubjectivity. Moreover, the pain-related prediction error signals in the PAG are functionally connected to the dmPFC (59). Thus, we postulate that the functional connectivity between the dmPFC and PAG should reflect the extent to which maternal defensive motivation can influence the representation of the child. In other words, we postulate that the dmPFC-PAG functional connectivity should modulate the maternal mirroring of the child as a function of parenting stress.

### The Abstract Level of Analysis to Link Intersubjectivity to Parenting Stress

To provide a theoretical relationship between interpersonal stress and the “over-mentalizing” and “under-coupling” problems at an abstract level of analysis, we postulate a dyadic active inference framework in a two-agent system, which will be elaborated in section Abstract Level of Analysis—Toward an Overarching Framework for Research on Intersubjectivity. Our framework is inspired by Karl Friston's Free Energy Principle (67, 68) and its application to stress (69). In brief, this framework postulates that in a two-agent system, stress ensues in a dyad when an agent's working model of the other agent in the system results in excessive prediction errors in a way that threatens the agent, and the stress worsens when the agent's preconceived working model of the other agent defies, rather than accommodates, the prediction errors. On the basis of this theoretical framework, we are led to postulate that when a mother shows symptoms of impaired intersubjectivity during mother-child interactions, she is at risk for excessive stress, and that her capacity to empathically mirror the child's actions and feelings may be compromised, reducing her sensitivity to the child's feelings, especially when the child's expressions are incongruent to the mother's preconceived working model/mental representation of her child. Thus, when she is stressed and/or in a negative mood, her mirroring of the child's joyful expressions may be diminished (i.e., stress-potentiated “under-coupling”), while her defensive reactions to the child's distressed expressions due to her preconceived working model may be exacerbated (i.e., stress-potentiated “over-mentalizing”). From prior work, we know that MP changes mothers' mental representations/working models toward less distorted/rigid/negative perceptions (28).

### The Empirical Level of Analysis in the Present Study

As the brain bases for “over-mentalizing” and “under-coupling” problems may be inferred through various experimental tasks in neuropsychiatric disorders (70), in the present study we employed a face and affect imitation task, namely Child Face Mirroring Task (CFMT) in the fMRI setting, which has been substantially modified from a previously published task (71). The CFMT involves pictorial displays of children's facial expressions, sorted in three independent factors, Child's Identity (Own Child and Other's Child), Emotions (Joy, Distressed, Ambiguous, and Neutral), and Task (Join, Observe, and React). In a full factorial design, each of the two children's pictures displayed four kinds of emotional expressions (Emotions), and all these pictures are repeated in three distinct conditions (Tasks): a face/affect mirroring condition (Join) and a non-mirroring control condition (Observe) to evoke strong and weak mother-child coupling, respectively, and, additionally, a React condition in which mothers respond to child faces as they normally would, to examine whether MP changes mothers' voluntary (un-instructed) responding. Results from two studies are reported here. In Study 1, in a sample of healthy mothers (*n* = 45), we examined the main effects of CFMT. In Study 2, we used CFMT in an randomized controlled intervention study where mother either receive the MP intervention (*n* = 19) or are in Control condition (*n* = 17), and measured maternal parenting stress at both pre- and post-treatment time points (T1 and T2) to identify potential neural mediators of MP effects on parenting stress.

Using CFMT, we computed a family of contrasts, namely Maternal Mirroring Response (MMR), to examine neural underpinning of own-child-specific maternal intersubjectivity. As these contrasts will be included in our predictions, we need to describe them before we prescribe the predictions. To isolate maternal neural responses in child-specific empathic mirroring across all emotions, we construed a MMR(all) contrast, i.e., Join[Own vs. Other's child's all emotions] vs. Observe[Own vs. Other's child's all emotions]. We also examine the contrast of positive vs. negative emotion in MMR, namely MMR(j-d), i.e., Join[Own vs. Other Child's Joyful vs. Distressed] vs. Observe[Own vs. Other Child's Joyful vs. Distressed]. The MMR(j-d) contrast approximately indicate the range of signed prediction errors, i.e., the range of MMR = MMR(j) - MMR(d), assuming that mirroring own child's joyful expression MMR(j) and distressed expression MMR(d) should elicit the maximum and minimum of prediction errors respectively in the brain regions that are sensitive to *signed prediction errors*. When these regions' sensitivity to signed prediction errors is diminished, e.g., MMR(j) is not different from MMR(d), then the range of MMR(j-d) should be no different from zero. The reasons for examining MMR(j-d) include: (1) as described above, the amygdala, NAc, and PAG are sensitive to *signed prediction errors* in emotional salience (reward or punishment), we postulate that these regions' MMR responses to positive (joyful) and negative (distressed) expressions may differ in the directions, e.g., relatively activated in MMR(joy) and deactivated in MMR(dis) for the amygdala and NAc, and vice versa for PAG; (2) the child's positive vs. negative facial expressions have been found to differentially activate maternal amygdala (72) as a function of unresolved stress (73), thus the maternal amygdala's sensitivity to the child's emotion during empathic mirroring may vary as a function of maternal stress. Taken together, the literature suggests that the maternal amygdala should be sensitive to MMR(j-d) and parenting stress may diminish the MMR(j-d) in the amygdala.

### Predictions

Based on the literature discussed above, we hypothesized that MP can reduce parenting stress by improving the mothers' working models of the child toward more flexible and positive perceptions, which can in turn improve maternal empathic mirroring of the child's joyful expressions (treating “under-coupling”) and can prevent the mothers' defensive reactions from coloring their mental representation of the child (treating “over-mentalizing”) during empathic mirroring. This hypothesis would be translated to the following group (MP vs. Control) by time (T1 vs. T2) interaction effects in the present study: We predict that, from T1 to T2, MP (vs. Control) will (1) reduce parenting stress measured with parenting stress index (PSI); MP (vs. Control) will rectify the “over-mentalizing” problem by (2) decreasing the *mentalizing system* activities during own-child-specific voluntary responding (React to Own vs. Other's Child); MP (vs. Control) will rectify the “under-coupling” problem by (3) increasing MMR(all) (own-child-specific empathic mirroring) in the *mirroring system* and by (4) increasing MMR(j-d) in the amygdala that mediates *signed prediction errors* of emotional salience. Because parenting stress can potentiate the “over-mentalizing” and “under-coupling” problems, we also predict that (5) the reduction in parenting stress will be associated with the reduction of the “under-coupling” problem, which can manifest as the association between the reduced parenting stress and increasing sensitivity to the *signed prediction errors* in the amygdala's MMR(j-d); (6) the reduction in parenting stress will be associated with the reduction of defensive “over-mentalizing,” which can manifest as the association between the reduced parenting stress and decreasing MMR(all)-dependent functional connectivity between the dmPFC (the sketchpad for child representation) and the PAG (the signals for defensive, fight-or-flight motivation). To summarize these predicted effects succinctly, we used non-parametric mediation analyses to identify potential neural mediators of MP treatment effects on parenting stress.

## Methods

### Ethics Approval Statement

The research reported in the current study was approved by the Institutional Review Board (IRB) at the University of Michigan, Ann Arbor, Michigan, USA. Informed consent from all participants was obtained. All research was performed in accordance with relevant IRB guidelines/regulations.

### Participants

All participants were recruited from low-income community clinics, primary care clinics, and/or community mental health centers. In Study 1, we examined brain responses during CFMT in a sample of healthy, unmedicated participants who underwent the CFMT (see below) for the first time (*n* = 45, age M = 31.78, SD = 7.62, child age M = 2.61, SD = 2.05). As MP's efficacy in reducing parenting stress has been established previously (27, 29), we conducted Study 2 to examine MP effects on intersubjectivity-dependent maternal brain responses and how these responses are associated with reduction in parenting stress. In Study 2, participants (*n* = 36) were randomly assigned to either MP treatment group (*n* = 19) or Control group (*n* = 17) and underwent the CFMT before (T1) and after (T2) MP or Control conditions, with about 14 weeks between scans. The participants in MP and Control groups differed slightly in their age [MP: M = 27.84, s.e. = 1.71; Control: M = 33.35, s.e. = 1.81, *F*_(1, 34)_ = 4.92, MSerror = 55.42, *p* = 0.033], but there was no group difference in the child age [MP: M = 2.25, s.e. = 0.40; Control: M = 3.09, s.e. = 0.42, *F*_(1, 34)_ = 2.08, MSerror = 3.06, *p* = 0.16] and number of offspring [MP: M = 1.63, s.e. = 0.19; Control: M = 1.65, s.e. = 0.20, *F*_(1, 34)_ = 0.003, MSerror = 0.66, *p* = 0.96]. There were three and five participants in MP and control groups, respectively, who were medicated with steady dosing anti-depressants across the study period. Nevertheless, we expected that the potential effects of medication would be canceled out for the following reasons: (1) medicated cases were in the minority and similarly distributed across MP and control groups (Chi-square *Z* = 0.963, *p* = 0.33), and (2) the repeated measures design controlled for the heterogeneity in medication status as participants are compared to their own baseline. As described further in the Supplementary Materials, removing medicated participants did not change results.

### Child Face Mirroring Task (CFMT)

**Figure 1:**
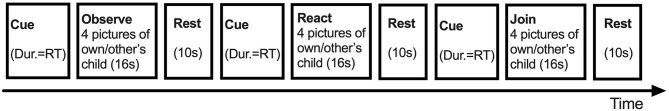
The design of Child Face Mirroring Task. Note that the task order in this figure did not represent the actual order. To protect the privacy, the pictures used in the task are not included here. However, examples of the task stimuli can be found in (71).

For the illustration of the task design, see Figure 1. In CFMT, participants were presented repeatedly with the same pictures of Own and Other Child in three conditions (Tasks), namely Observe, React, and Join. By design, the Observe Task should elicit the participant's unresponsive observation of face-like visual objects (i.e., “look-at-it,” a weak coupling condition), React should elicit the participant's usual, voluntary responses to the presented child, and Join should elicit the participant's empathic mirroring of the presented child (i.e., “empathize-you,” a strong coupling condition). The task instructions were presented to study participants as follows.

Observe: “You should simply observe the face on the screen. You should NOT make any face or generate any emotion. That is, BE an OBJECTIVE viewer of the faces. DO NOT FOLLOW any feelings depicted or caused by the face.”

React: “You should react to the emotion and expression of the child on the screen. You should imagine that you are the caregiver of the child on the screen. That is, you are REACTING to the emotions of the child on the screen as you normally would in your home.”

Join: “You should Join your own emotion with that of the emotion and expression of the child on the screen. You should empathize with the emotion depicted on the screen. That is, you are JOINING in the emotions of the child on the screen, with your OWN emotions.”

The three Tasks were presented block-by-block in a pseudo-random order. There were four pictures of a single child (one picture each for neutral, ambiguous, Distressed, and joyful expression), presented consecutively in a pseudo-random order, in each block (4 s each picture, 16 s per block). There were 4 blocks per Task for each of the Own and Other Child, with 10-s resting intervals between the blocks. To ensure the participants' wakefulness and readiness for the task, before each block, a single-word cue (“Observe,” “React,” or “Join”) was presented on the screen and participants pressed a button to indicate as soon as they were ready to perform the Task as instructed, without knowing which child's pictures would be presented. The reaction time in pressing the button was defined as Cue Period, reflecting the time required for a participant to be ready to perform the following task. The statistical analysis of the reaction time in Cue period is reported in Supplementary Materials.

### Task Stimuli

The participants provided all their child's pictures used in the study. The pictures of children unknown to the participants (Other's Child) were drawn from the in-house inventory. The lab staff standardized the stimuli qualities based on specific expressions (neutral, ambiguous, distressed, and joyful). We included these four kinds of expression following pioneering work in the field of parental neuroscience (71). Ratings of the child emotional expression images using Manikin Self-Assessment Scale (74) by four independent female raters confirmed the validity of the valence of the stimuli and the valence and arousal level were matched between the Own and Other Child's pictures, as described in Supplementary Materials and Supplementary Figure 1.

### MRI Procedures

Before each scan, the participants practiced CFMT to ensure their comprehension of the task and minimized effects due to stimuli novelty or learning. In each MRI scan, the participant was positioned in a supine orientation with her head positioned in a head coil. Visual stimuli were presented with E-Prime (PST, Inc., Pittsburgh, PA), via a goggle system and Nordic NeuroLab audio system. Behavioral responses were recorded by a button glove attached to the participant's right hand and linked to the E-Prime system. All fMRI scans were performed with a 3.0 Tesla Philips magnetic resonance imaging scanner using a standard 8-channel radiofrequency SENSE head coil with the following acquisition parameters: (1) A high-resolution T1 scan was acquired to provide precise anatomical localization (TR of 9.8 ms, TE = 459 ms, FA = 8°, FOV of 256 mm, slice thickness of 1.0 mm, 180 slices with 288 × 288 matrix per slice). (2) Two runs of T2^*^-weighted EPI sequence with BOLD (blood oxygenation level dependent) contrast (190 frames per run, TR = 2,000 ms, TE = 30 ms, FA = 90°, FOV = 220 mm, 42 contiguous axial slices, slice thickness = 2.8 mm with 64 × 64 matrix per slice, voxel size = 3.44 × 3.44 × 2.8 mm^3^) were acquired for whole-brain fMRI BOLD signal measures during the experimental task.

### MRI Data Processing and Analysis

For both Study 1 and 2, MRI data were pre-processed and analyzed using statistical parametric mapping software (SPM8; Welcome Department of Imaging Neuroscience, London UK). Five images at the beginning of each fMRI run were discarded to account for magnetic equilibrium. Slice timing correction was performed using a middle slice as a reference (slice 21). After slice time correction, images within each run were realigned to the mean image of the first run to correct for movement. Realigned functional images and structural image were spatially normalized using DARTEL method in SPM8. The normalized functional images were re-sliced to 2 × 2 × 2 mm voxels. Images were then spatially smoothed using a Gaussian filter with a full-width half-maximum value of 8 mm. All the images in the analyses and the figures are in neurological convention, with the left hemisphere presented at the left of an axial image.

#### First-Level Analysis

For both Study 1 and 2, following pre-processing, two first-level fixed effect General Linear Models (GLMs) were constructed to examine condition-dependent neural responses. The first model consisted of a matrix of regressors modeling 6 trial types (3 Tasks × 2 Child Identities: Observe Own, React Own, and Join Own and Observe Other's React Other's and Join Other's Child), in addition to a regressor for Cue periods (7 regressors total). The second model consisted of a matrix of regressors modeling each of four emotions (Neutral, Ambiguous, Distressed, and Joyful) for each of the six trial types, in addition to a regressor for Cue periods (25 regressors total). Additionally, a generalized Psychological-Physiological Interaction (gPPI) analysis (75) was performed to examine task-dependent functional connectivity with the dmPFC [81 voxels centered at MNI coordinates of [−2, 52, 20]] as the seed. The dmPFC seed cluster was selected because of its roles (as a “sketchpad” representing the child and as a hub whose functional connectivity) in mentalizing, described above, but also the only cluster identified in the conjunction of the Observe > Join main effect and the MP treatment group-by-time interaction effect on React to Own vs. Other Child, which is consistent with its role. Notably, because mathematically a variable's mean magnitude is independent of its correlations with other variables, using the dmPFC as the seed in gPPI analysis did not bias results, as the dmPFC was selected based on its magnitude in certain contrasts, which should be independent of the correlation analysis in gPPI. In gPPI, the physiological variable was estimated to be the average of the first eigenvariate of the BOLD time series of all voxels in the seed throughout the fMRI task. Then, this physiological variable was parsed into 7 condition-specific time-series based on the time window, defined by the onset and duration, of each condition convolved with the canonical hemodynamic response function, wherein the 7 conditions included three for Own Child (Observe Own, React Own, and Join Own), three for Other's Child (Observe Other's, React Other's, and Join Other's), and one for Cue periods. Then, the whole time series of the seed, the 7 condition-specific time series of the seed, the 7 conditions, and 6 motion parameters estimated during the realignment preprocessing were all entered as regressors (21 total) in a first level GLM.

##### Maternal Mirroring Contrasts

As part of the first-level analysis, we construed a family of contrasts related to maternal mirroring responses (MMR), which is defined as the capacity of the mother to empathically mirror her own child, given her current working model of her own child. There is a family of MMR contrasts based on the following linear combinations of the conditions in CFMT:

MMR(all): We construed MMR(all) as the contrast of [Join(Own Child's all expressions) – Observe(Own Child's all expressions)] – [Join(Other Child's all expressions) – Observe(Other Child's all expressions)] to isolate the mirroring process based on her current working model of child, while controlling for the general effects of looking at face-like visual objects (Join vs. Observe) and general empathic response to any child that is not specific to her own child (Own vs. Other's Child). The removal of the general empathic response is especially important here as the MP intervention aimed to specifically improve the mothers' working model of her child rather than their non-specific empathy.

MMR(j-d): We construed MMR(j-d) as the contrast of [Join(Own Child's Joy vs. Distress) – Observe(Own Child's Joy vs. Distress)] – [Join(Other Child's Joy vs. Distress) – Observe(Other Child's Joy vs. Distress)]. This contrast measured a signed value (vector) of the difference between positive and negative valence in MMR.

MMR(joy/dis/amb/neu): To examine MMR in each kind of emotional expression separately, we construed MMR(joy/dis/amb/neu) as the contrast of [Join(Own Child's joy/dis/amb/neu) – Observe(Own Child's joy/dis/amb/neu)] – [Join(Other Child's joy/dis/amb/neu) – Observe(Other Child's joy/dis/amb/neu)] in only the joy, distressed, ambiguous, or neutral expressions, respectively.

Notably, because all emotional expressions were presented in a random order, the MMR for each emotional expression is directly related to the prediction errors to that expression with reference to the implicit expectation built up during the preceding expression as baseline, which may be based on any other types of expressions. Because MMR(j) should always elicit a response that is more positive in valence than any of its preceding baseline, be it MMR(d), MMR(n), or MMR(a), and, likewise, MMR(d) should always elicit a response that is more negative in valence than any of its preceding baseline, be it MMR(j), MMR(n), or MMR(a). Thus, logically, MMR(j) should elicit the most positive possible prediction errors (the maximum of better-than-expected prediction error) and MMR(d) should elicit the most negative possible prediction errors (the minimum of worse-than-expected prediction error), and therefore MMR(j) - MMR(d) approximates the range of MMRs, i.e., range(MMR) = max(MMR) - min(MMR). Supposedly if a region's sensitivity to signed prediction errors is diminished, e.g., MMR(j) is not different from MMR(d), then the range of MMRs, i.e., MMR(j-d), should be no different from zero. Thus, MMR(j-d) is an index of the sensitivity to *signed prediction errors*. Note that a region that is activated in MMR(joy) but deactivated in MMR(dis) means that the region is sensitive to reward-like prediction errors, resulting in a positive MMR(j-d) in the region, e.g., the NAc (55, 56) and amygdala (57). Conversely, a region that is deactivated in MMR(joy) but activated in MMR(dis) means that the region is sensitive to punishment-like prediction errors, resulting in a negative MMR(j-d) in the region, e.g., PAG (59) and amygdala (57, 58). In other words, MMR(j-d) is a vector indicating the sensitivity of signed prediction errors in a region.

Also, general empathic responses to unknown child were removed from the MMRs to isolate the changes in the mother's own-child-specific empathic responses, because the mothers already have specific preconceived working models of their child, which is believed to be improved by MP. This contrast thus isolates the responses that are specific to the very mother-child dyad, i.e., the primary focus of the MP dyadic intervention. This is consistent with the notion that intersubjectivity is best investigated in a dyadic framework involving first-person and second-person perspectives (76).

#### Second-Level Analysis

The contrasts of interest from the first level GLMs were submitted to six second-level random effect GLMs. (1) **CFMT effects:** To establish the effects of the novel CFMT at T1, we examined the main effects of Task, Child and the Task by Child interaction, with the age of the Own Child as a covariate, to control for the children's varying social developmental stages that may influence the maternal responses (77). (2) **MP treatment effects**: In Study 2, we examined MP treatment (vs. Control) by Time interaction effects on MMR(all), MMR(j-d), and React of Own vs. Other Child (i.e., mothers' voluntary response to own child). (3) **Mediation analysis**: To summarize results according to our predictions, we performed X-M-Y mediation analysis, using the MP vs. Control as a categorical predictor (*X*), T1-to-T2 changes in parenting stress as outcome (*Y*), and testing three potential intersubjectivity-dependent brain mediators: T1-to-T2 changes in the differential responses in MMR(all) (as *M*_1_), the MMR(all)-dependent gPPI with the dmPFC seed (as *M*_2_), and MMR(j-d) (as *M*_3_). In this analysis, we first identified candidates of potential mediators showing significant effects on both X-M (Path-*a*) and M-Y (Path-*b*), and then submitted the three potential mediators to mediation analysis, controlling for the child age, to compute the 95% confidence interval of indirect effects between X and Y, based on the non-parametric bootstrapping method with 5,000 times of sampling.

Unless specified otherwise, all the second-level models were tested with whole-brain correction at family-wise error (FWE) = 0.05. Besides whole brain analysis, we performed Bonferroni family-wise small volume corrections (s.v.c.), separately, in the subcortical regions known to modulate maternal behaviors (52, 53, 78), with their masks derived from the wfu_pickatlas toolbox (79), including amygdala [as defined in wfu_pickatlas' AAL domain (80)], periaqueductal gray (PAG) (a 8 mm × 6 mm × 8 mm box centered at [0, −28, −12] in MNI coordinates), hypothalamus [as defined in wfu_pickatlas' TD Brodmann areas+ domain (79)], midbrain [as defined in wfu_pickatlas' TD Lobes domain (79)], nucleus accumbens (NAc) [a 18 mm × 8 mm × 10 mm box centered at [0, 10, −14] in MNI coordinates], and striatum [putamen, as defined in AAL (80)].

### Procedures in Study 2 Only

#### Mom Power (MP) Parenting Intervention

MP is a relationship-based parenting group therapy designed to promote positive parenting, reflective capacity, parental mental health and secure child-parent relationships. The curriculum rests on five core pillars paralleling the Strengthening Families Protective Factors Framework (81): (1) attachment-based parenting education, (2) self-care, (3) mother-child interaction practice, (4) social support, and (5) connection to resources. For a detailed description of the intervention, please see Supplementary Materials. Women randomized to the MP treatment arm received the 13-session manualized MP parenting intervention (3 individual sessions and 10 group sessions) led by community clinicians trained via a 3-day in person course with model developers. Groups were co-facilitated by two interventionists, at least one being a Master's level clinician, and fidelity was monitored via weekly reflective supervision as well as video review of 20% of all sessions using a fidelity monitoring scale (82). Fidelity was formally assessed using a 5-point Likert scale (5 = highest fidelity) for both content (i.e., fidelity to manual content) and framework (i.e., fidelity to the therapeutic framework dedicated to creating a therapeutic milieu based in attachment theory and trauma informed care). Fidelity was found to be excellent across clinicians for both content (*M* = 4.02, *SD* = 0.72) and framework (*M* = 3.85, *SD* = 0.69).

##### Control Group

Mothers randomized into the Control group received two individual sessions (pre/post) and 10 weekly mailings of the MP curriculum content without the in-person group components. Mailings included a pre-stamped post card for mothers to send back indicating that the week's material had been read. Participants were compensated $5 for each postcard returned, and an additional $15 if they returned 7/10 postcards.

#### Self-Reported Measure

##### Parenting Stress Index (PSI)

The PSI (83) is a 36-item questionnaire designed to measure levels of parenting stress and previously found to be valid, reliable, and sensitive to change across diverse populations (84). The PSI yields a PSI Total Score that was used for present analyses, which has been shown to have excellent internal consistency (Cronbach's alpha = 0.92) and good test-retest reliability (Intraclass Correlation Coefficients = 0.78) (85).

### Non-fMRI Analysis and Results

We tested the group differences in demographic variables and the MP treatment effects on parenting stress (as indexed by PSI) in GLMs, using SPSS v.24 (IBM Corp. Armonk NY). We also performed the non-parametric mediation analysis based on the bootstrapping of 5,000 times of re-sampling, with a covariate of Own Child's age, using the macro of PROCESS (86) in SPSS v.24 (IBM Corp. Armonk NY). Due to the space limit, the results of these non-MRI analyses are described in Supplementary Materials and Supplementary Figure 1 (Independent raters' rating on the stimuli), Supplementary Figure 2 (MP effects on PSI), Supplementary Figure 3 (Cue period of CFMT), and Supplementary Figure 4 (robustness check after removing medicated participants in Study 2).

## Results

### Study 1: Child Face Mirror Task Effects

**Figure 2:**
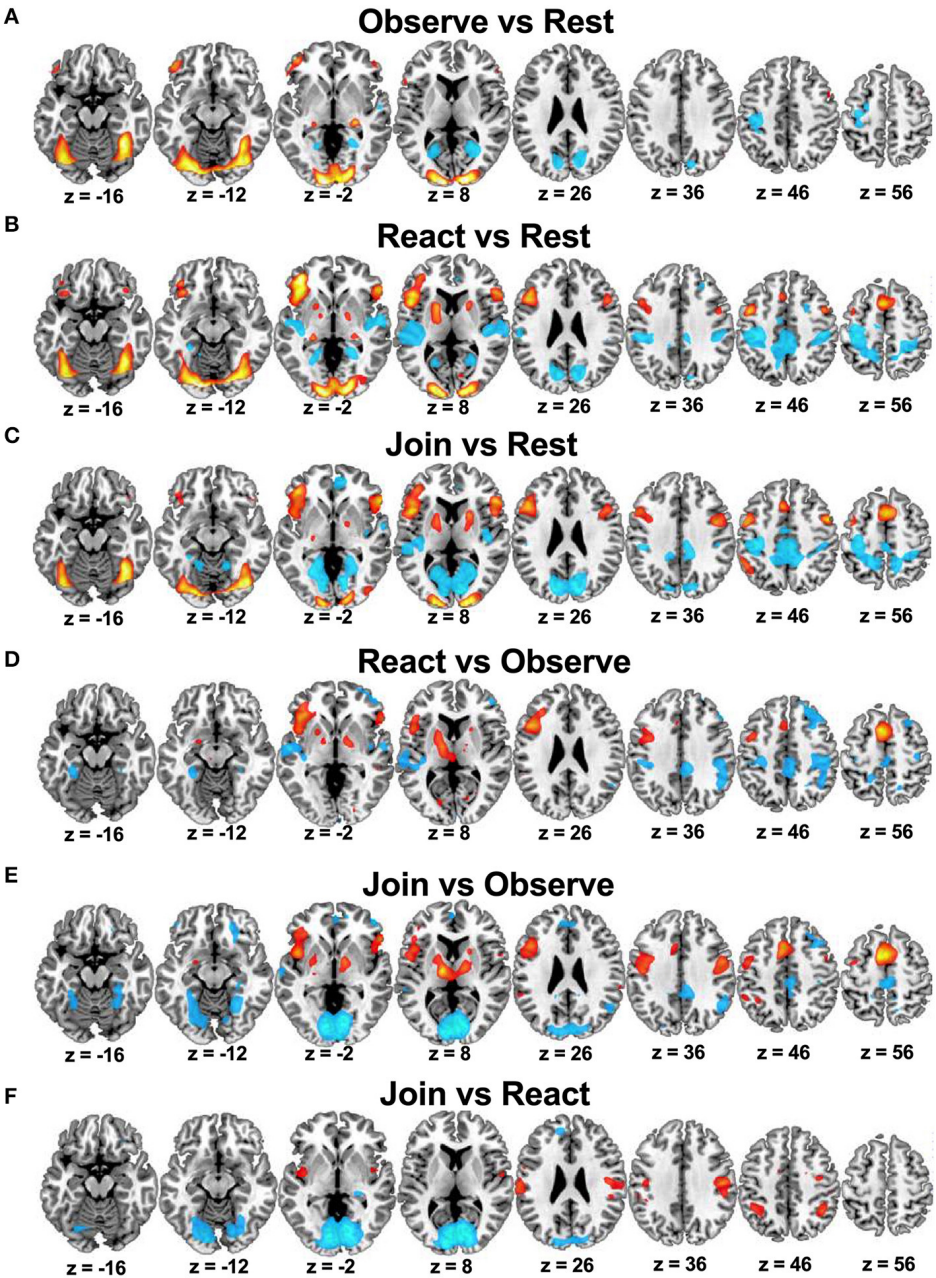
Whole brain results in the reference sample of healthy mothers (*n* = 45) from Study 1: Brain regions that were relatively activated (in hot color) or deactivated (in cool color) in pairwise Task contrasts of Observe vs. Rest **(A)**, React vs. Rest **(B)**, Join vs. Rest **(C)**, React vs. Observe **(D)**, Join vs. Observe **(E)**, and Join vs. React **(F)**.

**Figure 3:**
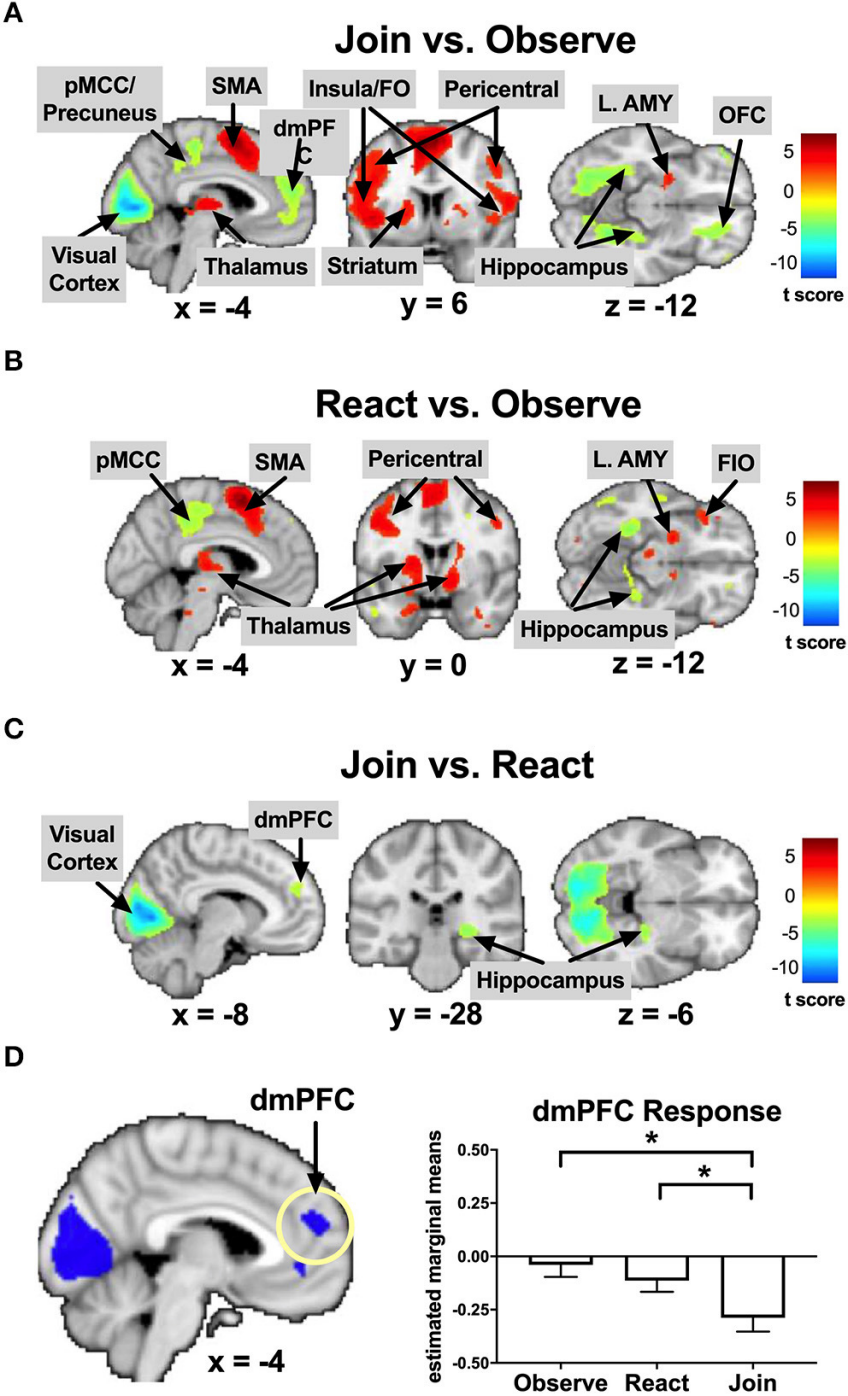
Key results in the reference sample of healthy mothers (*n* = 45) from Study 1: Brain regions that were relatively activated (in hot color) or deactivated (in cool color) in pairwise Task contrasts of Join vs. Observe **(A)**, React vs. Observe **(B)**, and Join vs. React **(C)**. The dmPFC was inhibited in Join vs. Observe and Join vs. React, with the bar charts for each Task's mean (±s.e.) separately **(D)**. pMCC, posterior middle cingulate cortex; SMA, supplemental motor area; dmPFC, dorsomedial prefrontal cortex; Insula/FO, insula/frontal operculum; L. AMY, left amygdala; OFC, orbitofrontal cortex; FIO, frontal inferior orbital. *p < 0.05.

We first report the results of primary main effects in CFMT. The main effects of Tasks (Observe, React, and Join vs. Rest) and the pairwise planned contrasts (React vs. Observe, Join vs. Observe, and Join vs. React), pooling across both children, are summarized in Table 1 and Figure 2, with the key brain regions depicted in Figure 3. As expected, all three Tasks activated face-related processing in visual cortex and fusiform face area (FFA). Interestingly, the neural responses in some of these visual processing areas were attenuated in both Join vs. Observe and React vs. Observe contrasts. Conversely, both Join vs. Observe and React vs. Observe contrasts activated brain regions involved in the *mirroring system* (32), including pericentral, insular, frontoparietal cortices, and thalamus, and the salience network (49), including striatum, and amygdala.

**Table 1:** Task main effects (vs. rest).

		**MNI coordinates**			**No. of voxels**	
**Brain region**	**Side**	**X**	**Y**	**Z**		***Z*-score**
**Observe** **>** **rest**						
Occipital lobe	L	−12	−94	−8	5,373	7.13
	R	14	−96	8		7.05
Hippocampus	R	26	−26	−2	14	5.21
Inferior frontal gyrus (IFG)	L	−44	50	−6	57	5.19
**React** **>** **rest**						
Occipital lobe	L	−12	−90	−10	2,544	7.45
	R	18	−86	−8		7.43
IFG/middle frontal gyrus (MFG)	L	−40	40	−4	1,020	6.79
(including fontal operculum, FOp)	L	−48	14	4	(81)	6.13
	R	54	28	2	142	5.52
Supplemental motor area (SMA)	R/L	−4	10	60	246	6.05
Pericentral gyrus	L	−46	2	46	46	5.29
Lentiform nucleus (pallidum/putamen)	L	−44	50	−6	57	5.19
**Join** **>** **rest**						
Occipital lobe	R	18	−86	−8	1,031	6.91
	L	−36	−58	−22	623	6.44
FOp	L	−46	14	4	178	5.96
SMA	R/L	6	8	62	340	5.65
IFG	L	−42	38	0	192	5.60
Pericentral gyrus	R	48	4	46	95	5.36
	L	−48	2	48	54	5.17
MFG	L	−48	20	28	121	5.32
Lentiform nucleus (pallidum/putamen)	R	22	10	8	6	4.67

As summarized in Table 2 and Supplementary Figure 5, the brain regions that were conjunctively implicated in both Join vs. Observe and Join vs. React contrasts included the bilateral pericentral cortices and left inferior parietal lobule (IPL), which were more activated in Join than the other two Tasks, and the occipital and lingual cortices, right hippocampus, and the dmPFC, which were less activated in Join than the other Tasks (also depicted in Figure 3D).

**Table 2:** Task main effects in pairwise contrasts^*^.

		**MNI Coordinates**			**No. of voxels**	
**Brain region**	**Side**	**X**	**Y**	**Z**		***Z*-score**
**Join** **>** **observe**						
SMA	R/L	−6	6	58	2,252	5.83
Thalamus (including hypothalamus)	L	−14	−14	10	483	5.59
	R	12	−10	4	340	4.85
FOp/insula	L	−44	10	2	1,979	5.45
	R	56	28	−4	788	4.68
Pericentral gyrus	R	50	0	38	647	4.52
	L	−36	2	38	1,336	4.38
Lentiform nucleus (pallidum/putamen)	R	14	−4	2	336	4.71
	L	−14	−6	2	513	4.27
Inferior parietal lobule (IPL)	L	−36	−48	44	407	3.68
Amygdala	L	−24	−2	−12	25	3.33
**Observe** **>** **join**						
Occipital lobe (cuneus/calcarine)	R	10	−80	4	7,293	7.72
(including parahippocampal gyrus)	L	−8	−82	2		7.40
Precuneus/middle cingulate cortex (MCC)	R/L	4	−26	50	1,885	4.65
Temporoparietal junction (TPJ)/angular gyrus	R	52	−52	36	693	4.59d
Dorsomedial prefrontal cortex (dmPFC)	R/L	−6	50	18	1,483	4.15
IFG/Fontal inferior orbital (FIO)	R	50	48	−2	235	4.12
Orbitofrontal cortex (OFC)	R	26	36	−12	211	3.84
MFG (BA 8)	R	34	20	46	569	3.80
**React** **>** **observe**						
SMA	R/L	−6	10	58	1,353	5.87
FO/MFG/IFG/precentral	L	−44	12	2	4,287	5.09
(including thalamus/lentiform nucleus)						
Lentiform nucleus	R	14	−4	−6	436	4.36
(including thalamus)	R	18	−16	12	(112)	3.28
Pericentral gyrus	R	50	0	38	647	4.52
	L	−36	2	38	1,336	4.38
IFG	R	54	26	0	303	4.11
FIO/temporal pole	L	−24	18	−24	318	3.99
**Observe** **>** **react**						
Superior temporal gyrus (STG)	L	−56	−10	−2	1,198	4.83
	R	58	−8	−2	201	4.02
Parietal lobe/postcentral	R	48	−28	42	2,293	4.78
MCC/paracentral lobule	R/L	6	−32	40	2,397	4.46
MFG (BA 8)	R	24	32	44	1,070	4.39
IFG/Fontal inferior orbital (FIO)	R	48	50	2	428	4.15
Hippocampus, posterior	L	−28	−40	−12	275	4.11
**Join** **>** **react**						
None						
**React** **>** **Join**						
Occipital lobe (cuneus/calcarine)	R/L	12	−78	8	6,977	>15
dmPFC	R/L	−14	50	28	232	3.64

* *Whole brain corrected at false-discovery rate (FDR) = 0.05*.

The main effects of Child (Own vs. Other Child) are summarized in Supplementary Table 1. The neural responses in the occipital, precuneus, angular gyrus, and FIO cortices were greater in Own than Other Child. These regions are largely involved in autobiographical memory, thus consistent with their roles in the *mentalizing system*.

Since there were some Task-by-Child-interaction effects, described below, we examined the simple main effects of Own vs. Other Child in each Task separately (Supplementary Table 1). For Observe, we found that the Own vs. Other Child in this Task elicited differential neural responses in the visual face processing areas (FFA) and autobiographical memory-related regions (i.e., FIO, temporal poles and hippocampus), and cognitive regulatory regions (right dorsolateral prefrontal cortex (dlPFC) and supplemental motor area (SMA), which were more active in the main effects of Join (>Observe) and React (>Observe), indicating that the mothers automatically engaged the Own Child with more autobiographical and interactive responses than they did in Observe of Other Child, despite that the task instruction of Observe explicitly discouraged such active child-oriented responses. For React, the Own vs. Other Child elicited differential responses in the subcortical regions, including the thalamus, hypothalamus, striatum, hippocampus, and midbrain, suggesting the mothers responded to Own Child with greater maternal motivation than they did to Other Child. For Join, there were no Own vs. Other Child differences in any regions.

The planned tests related to Task-by-Child interaction effects [including MMR(all) and MMR(j-d)] are summarized in Supplementary Table 2. For MMR(all), we found that the precuneus and fusiform gyrus showed greater Own > Other differential responses in Observe than in Join (Observe > Join)—which is an inverse MMR(all)—suggesting that the Join, as compared to Observe, *reduced* the face processing, mediated by the fusiform gyrus (87), and narrative thinking processing, mediated by the precuneus (41), related to Own Child. We also found that the midbrain, striatum/extended amygdala, and hypothalamus showed greater Own > Other differential responses in React than in Join—suggesting that the own-child-specific maternal motivation responses were stronger in the React than Join. For MMR(j-d), we found that the left amygdala was associated with MMR(j-d) (MNI coordinates: [−26, 2, −24], 15 voxels, *Z* = 3.17, *p* = 0.021 s.v.c.).

### Study 2: MP Treatment Effects

We predicted MP treatment effects on parenting stress, maternal voluntary mirroring (probed in the React Condition) and maternal mirroring responses [MMR(all) and MMR(j-d)]. For parenting stress, we found that MP, relative to Control, showed lower PSI total scores at T2 (see Supplementary Materials). We examined MP Treatment effects by testing Group-by-Time interaction effects on three own-child-specific contrasts, i.e., React to Own vs. Other Child, MMR(all), and MMR(j-d).

**Figure 4:**
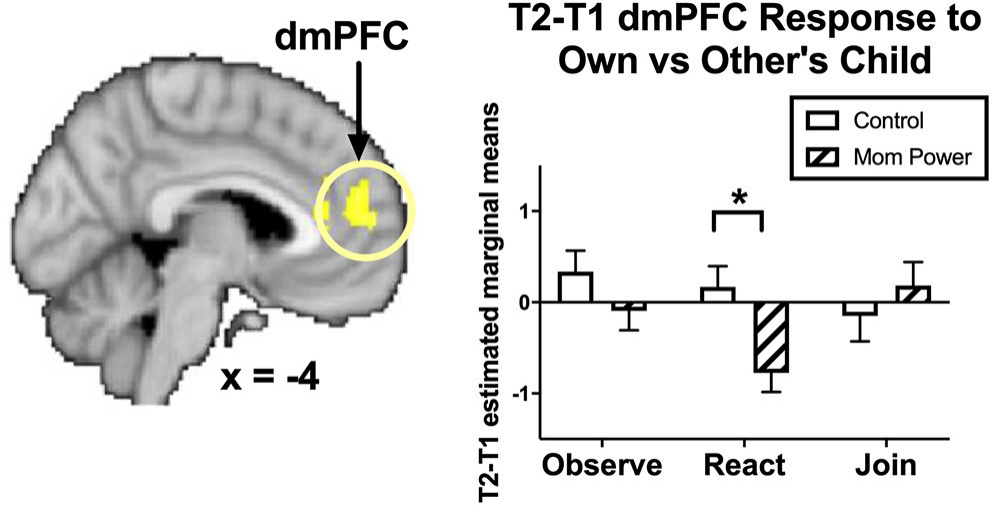
From T1 to T2 in the clinical study sample (Study 2), MP, relative to Control, showed greater inhibition in the dmPFC during React to Own vs. Other Child, with the bar charts for each Task's mean (±s.e.) separately. *p < 0.05.

For React to Own vs. Other Child, we found that, from T1 to T2, MP, relative to Control, decreased React to Own (vs. Other) Child responses in the dmPFC ([−8, 54, 14], 887 voxels, *Z* = 3.08, *p* = 0.009, whole brain cluster-level FWE corrected, Figure 4).

**Figure 5:**
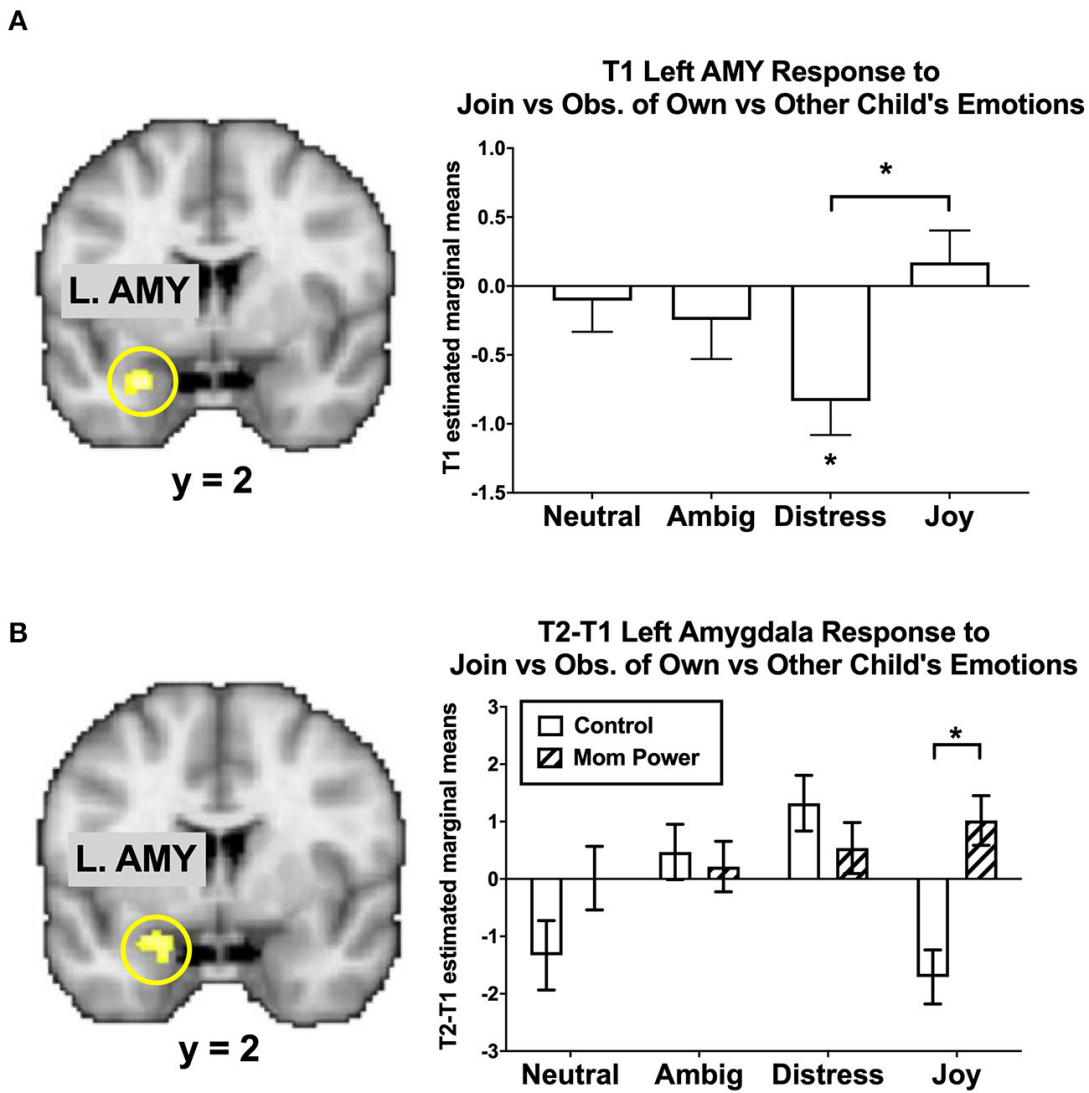
The left amygdala's MMR(all) [Join[Own vs. Other Child] vs. Observe[Own vs. Other Child]] differential responses was activated in Joyful vs. Distressed expression, while it was inhibited in the Distressed expression in the reference sample, with the bar charts of each expression's mean (±s.e.) separately **(A)**. From T1 to T2 in the clinical study sample, MP, relative to Control, showed greater activation in the Joyful expression in the Join[Own vs. Other Child] vs. Observe[Own vs. Other Child], with the bar charts for each expression's mean (±s.e.) separately **(B)**. *p < 0.05.

As the amygdala mediated MMR(j-d) in the reference sample (Figure 5A), there were several Group-by-Time interaction effects on the amygdala as follows. We found that MP, relative to Control, increased the MMR(j-d) in the left amygdala ([−24, −2, −18], 24 voxels, *Z* = 2.87, *p* = 0.046 s.v.c., Figure 5B), in which the differential response to own child's Joy expression was increased in MP, but decreased in Control, mothers.

These results suggested that MP mothers, relative to Control, developed stronger capacity not only to activate the left amygdala in response to own child's joyful faces when they were instructed to mirror the children's emotions in the Join condition, but also to inhibit the own-child-specific neural responses in the dmPFC (Figure 4) during their voluntary mirroring responses to their own child in the React condition.

**Figure 6:**
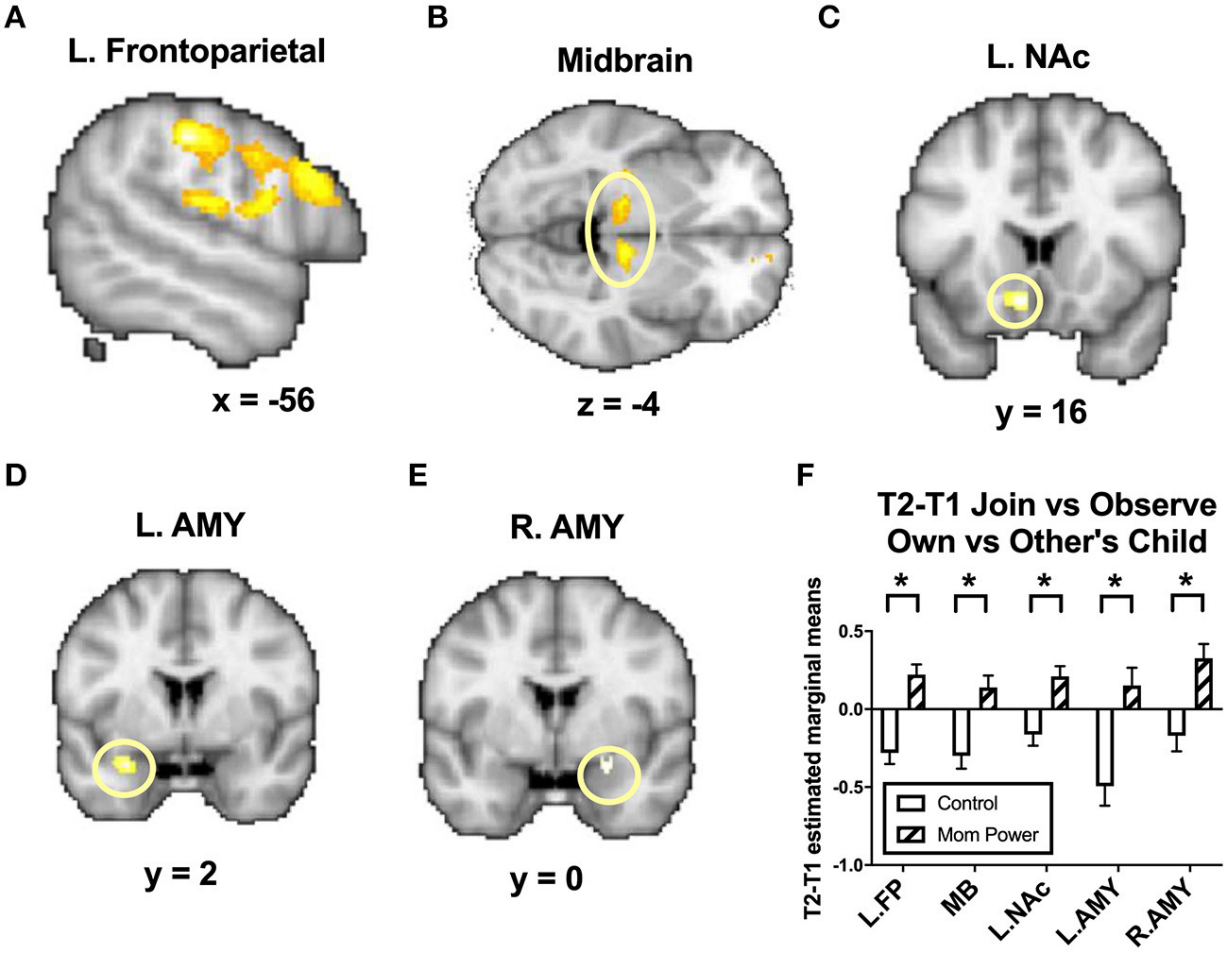
From T1 to T2 in the clinical study sample, MP, relative to Control, showed greater differential responses of MMR(all) [Join[Own vs. Other Child] vs. Observe[Own vs. Other Child]] in the left frontoparietal regions **(A)**, midbrain **(B)**, left nucleus accumbens (NAc) **(C)**, left and right amygdala (AMY) **(D,E)**, with the bar charts of each region's mean (±s.e.) **(F)**. *p < 0.05.

For MMR(all), from T1 to T2, MP, relative to Control, increased the MMR(all) in the left frontoparietal regions including the parietal/postcentral ([−56, −26, 42], 357 voxels, *Z* = 4.22, *p* = 0.001, whole brain cluster-level FWE corrected) and dorsolateral prefrontal cortex ([−56, 16, 28], 706 voxels, *Z* = 3.48, *p* = 0.001, whole brain cluster-level FWE corrected, Figure 6A), midbrain ([10, −20, −4], 124 voxels, *Z* = 3.55, *p* = 0.049 s.v.c., Figure 6B), left NAc ([−8, 16, −12], 26 voxels, *Z* = 3.37, *p* = 0.013 s.v.c., Figure 6C), left amygdala ([−28, 2, −22], 12 voxels, *Z* = 3.18, *p* = 0.021 s.v.c., Figure 6D), and, marginally, right amygdala ([24, 0, −16], 18 voxels, *Z* = 2.85, *p* = 0.057 s.v.c., Figure 6E).

To examine the results in elementary conditions, such as specific tasks and emotions, we unpacked the elements involved in the Figure 5B in the Supplementary Figure 6. Similarly, we also unpacked the elements involved in the Figure 6F in the Supplementary Figure 7.

#### Mediation Analysis

To summarize succinctly the results reported above, we utilized mediation analysis to identify potential mediators of MP effects on reducing parenting stress. We performed mediation analysis using the treatment group as the categorical predictor (*X*), T1-to-T2 changes in parenting stress (dPSI) as the outcome (*Y*), and T1-to-T2 changes in MMR(all) and MMR(j-d) as potential mediators (*M*'s). For each of the i'th potential meditator (*M*_*i*_), we denote the *X-M* path as Path-*a*_*i*_, the *M-Y* path as Path-*b*_*i*_, the indirect effect as Path-*a*_*i*_*b*_*i*_, and the direct effects of *X* on *Y* as Path-*c'*_*i*_.

**Figure 7:**
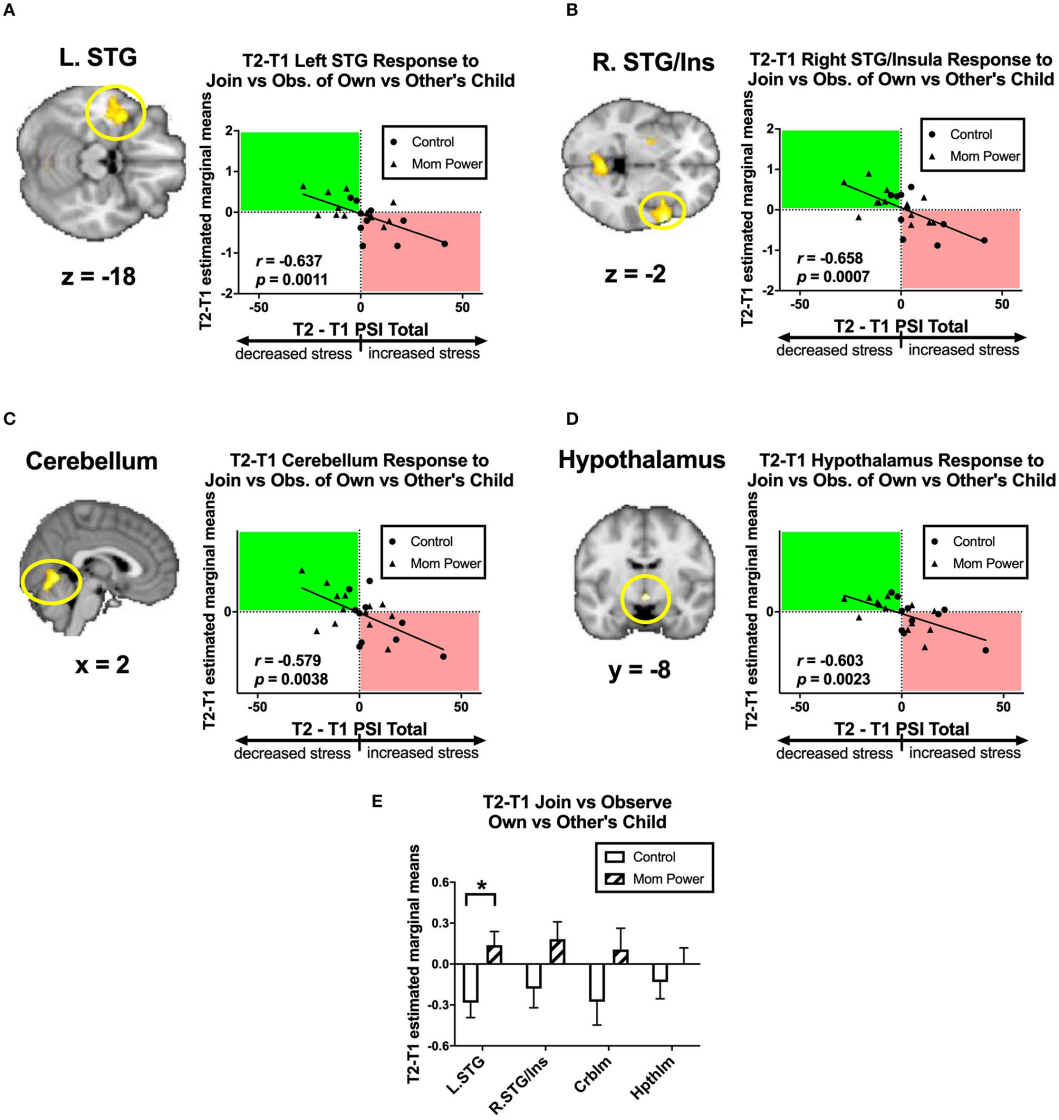
From T1 to T2 in the clinical study sample, the T2-T1 differences in parenting stress index (dPSI) were negatively associated with the concomitant increases in the MMR(all) [Join[Own vs. Other Child] vs. Observe[Own vs. Other Child]] differential responses in the left superior temporal gyrus (STG) **(A)**, right STG/insula **(B)**, cerebellum **(C)**, and hypothalamus **(D)**, each with the dPSI depicted on the x-axis, against the T2-T1 difference in the region's differential response on the y-axis, in the scatter plots. The Pearson's correlation *r* scores and *p*-values are embedded in the plots. The bar charts of each region's mean (±s.e.) are depicted in **(E)**. *p < 0.05.

Firstly, we identified candidates of potential mediators by regressing the T1-to-T2 changes in the MMR(all) against dPSI, controlling for the baseline PSI at T1. We found that the T1-to-T2 reduction of parenting stress was associated with the T1-to-T2 MMR(all) increases in the left superior temporal gyrus (STG) ([−40, 4, −18], 563 voxels, *Z* = 3.56, *p* = 0.034 whole brain cluster-level FWE corrected, Figure 7A), right STG ([60, 10, −2], 662 voxels, *Z* = 4.30, *p* = 0.016 whole brain cluster-level FWE corrected, Figure 7B), cerebellum ([2, −62, −4], 622 voxels, *Z* = 3.86, *p* = 0.022 whole brain cluster-level FWE corrected, Figure 7C), and hypothalamus ([0, −8, −10], 12 voxels, *Z* = 3.05, *p* = 0.049 s.v.c., Figure 7D). Among these regions, the MP vs. Control difference in the MMR(all) was significant only in the left STG [*F*_(1, 21)_ = 7.61, *MS*_*error*_ = 0.12, *p* = 0.012, Figure 7E]. Thus, we identified the MMR(all) in the left STG as the first potential mediator, denoted as *M*_1_.

**Figure 8:**
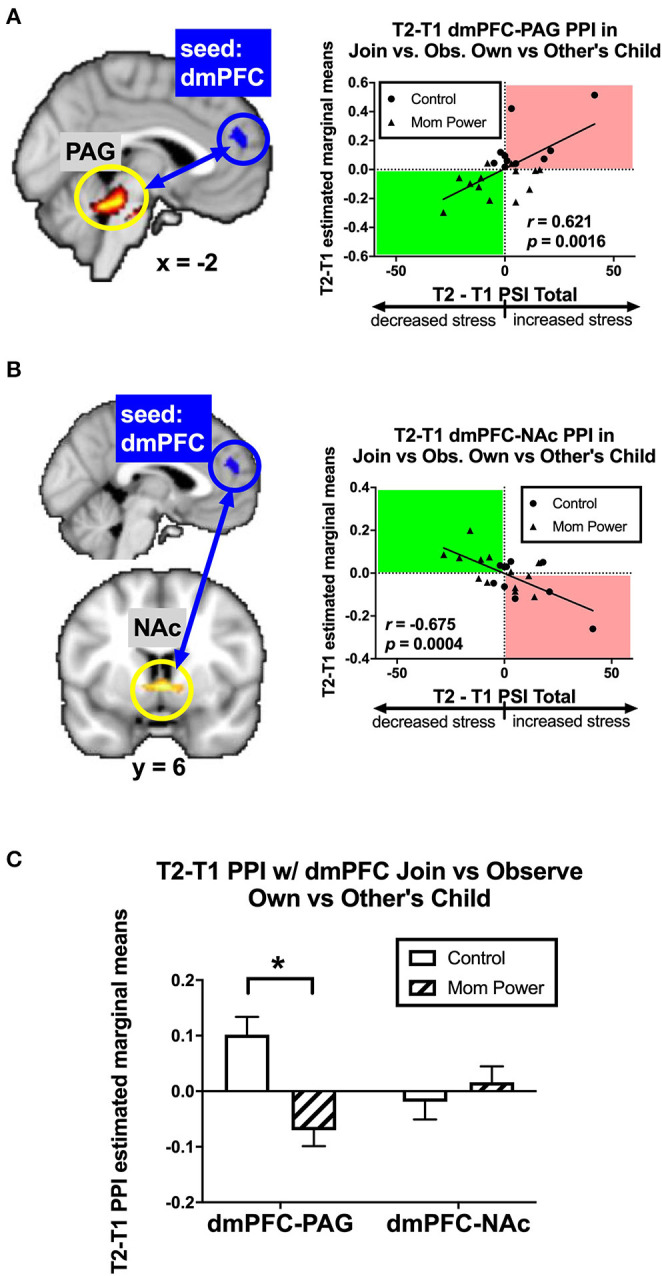
From T1 to T2 in the clinical study sample, the T2-T1 differences in parenting stress index (dPSI) were positively and negatively associated with the concomitant increases in the MMR(all) [Join[Own vs. Other Child] vs. Observe[Own vs. Other Child]] differential functional connectivity [MMR(all)-dependent PPI] between the dmPFC and PAG **(A)** and that between the dmPFC and NAc **(B)**, respectively, each with the dPSI depicted on the x-axis, against the T2-T1 difference in the region's differential response on the y-axis, in the scatter plots. The Pearson's correlation *r* scores and *p*-values are embedded in the plots. The MP vs. Control differed in the MMR(all)-dependent PPI between dmPFC and PAG, but not that between dmPFC and NAc, with the bar charts of each region's mean (±s.e.) depicted in **(C)**. *p < 0.05.

Secondly, we identified candidates of potential mediators by regressing the T1-to-T2 changes in the dmPFC's MMR(all)-dependent psychological-physiological interaction (PPI) against dPSI, controlling for the baseline PSI at T1. We found that the T1-to-T2 increases in parenting stress was associated with the T1-to-T2 increases in the MMR(all)-dependent PPI between the dmPFC seed and the PAG ([−2, 32, −20], 178 voxels, *Z* = 4.36, *p* = 0.002 s.v.c., Figure 8A); conversely, the T1-to-T2 reduction in parenting stress was associated with T1-to-T2 increases in the MMR(all)-dependent PPI between the dmPFC seed and bilateral NAc ([6, 6, −4], 40 voxels, *Z* = 3.39, *p* = 0.020 s.v.c., Figure 8B). Among these PPI results, the MP vs. Control group difference in the MMR(all)-dependent PPI was significant only in the dmPFC-PAG [*F*_(1, 21)_ = 14.99, *MS*_*error*_ = 0.10, *p* = 0.001, Figure 8C]. Thus, we identified the MMR(all)-dependent PPI between the dmPFC-PAG as the second potential mediator, denoted as *M*_2_.

**Figure 9:**
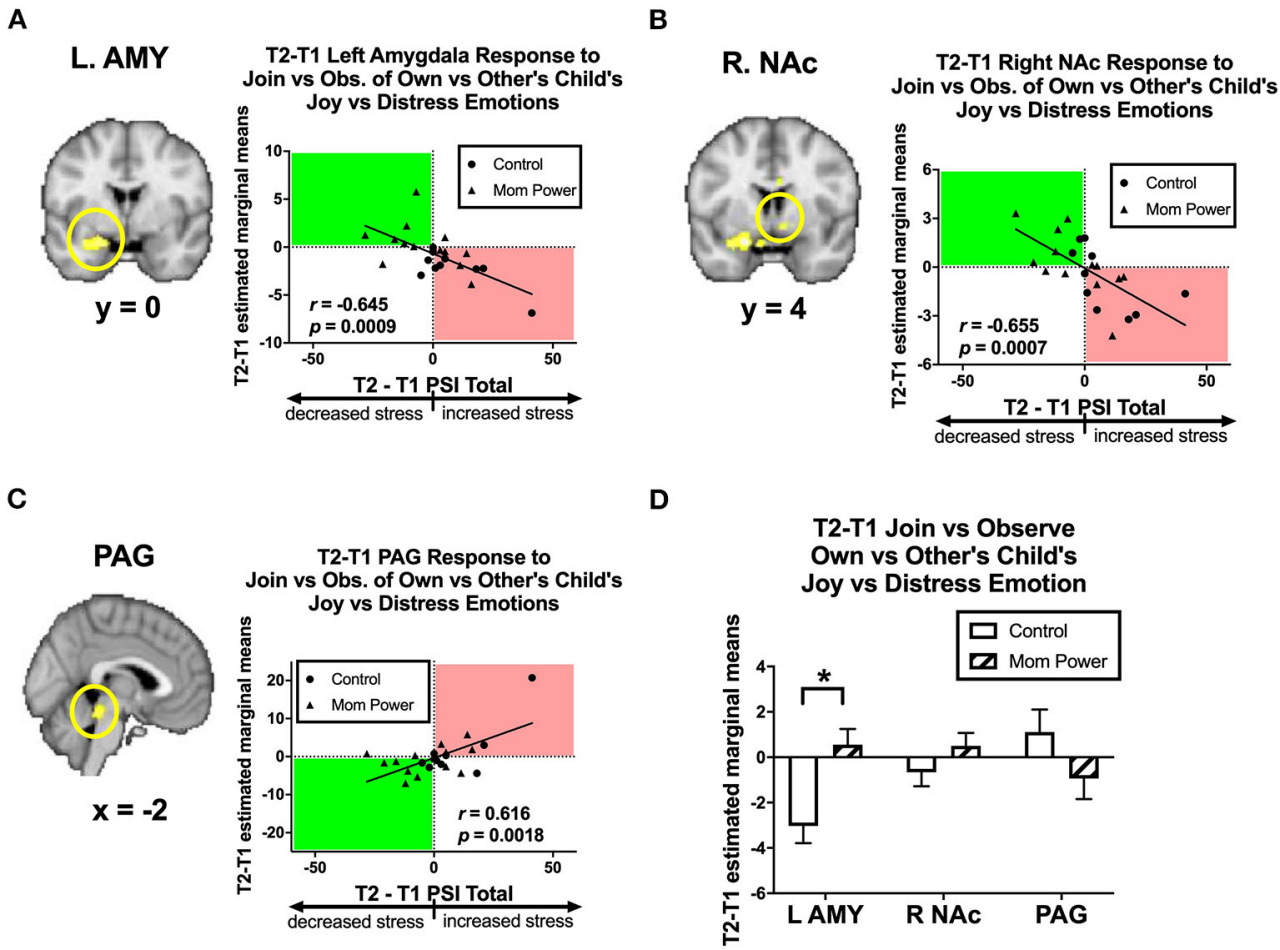
From T1 to T2 in the clinical study sample, the T2-T1 differences in parenting stress index (dPSI) were negatively associated with the concomitant increases in the MMR(j-d) [Join[Own vs. Other Child's Joyful vs. Distressed] vs. Observe[Own vs. Other Child's Joyful vs. Distressed]] differential responses in the left amygdala **(A)** and right NAc **(B)**, but they were positively associated with that in the PAG **(C)**, each with the dPSI depicted on the x-axis, against the T2-T1 difference in the region's differential response on the y-axis, in the scatter plots. The Pearson's correlation *r* scores and *p*-values are embedded in the plots. The MP vs. Control differed in the MMR(j-d) in the left amygdala, but not the right NAc and PAG, with the bar charts of each region's mean (±s.e.) depicted in **(D)**. *p < 0.05.

**Figure 10:**
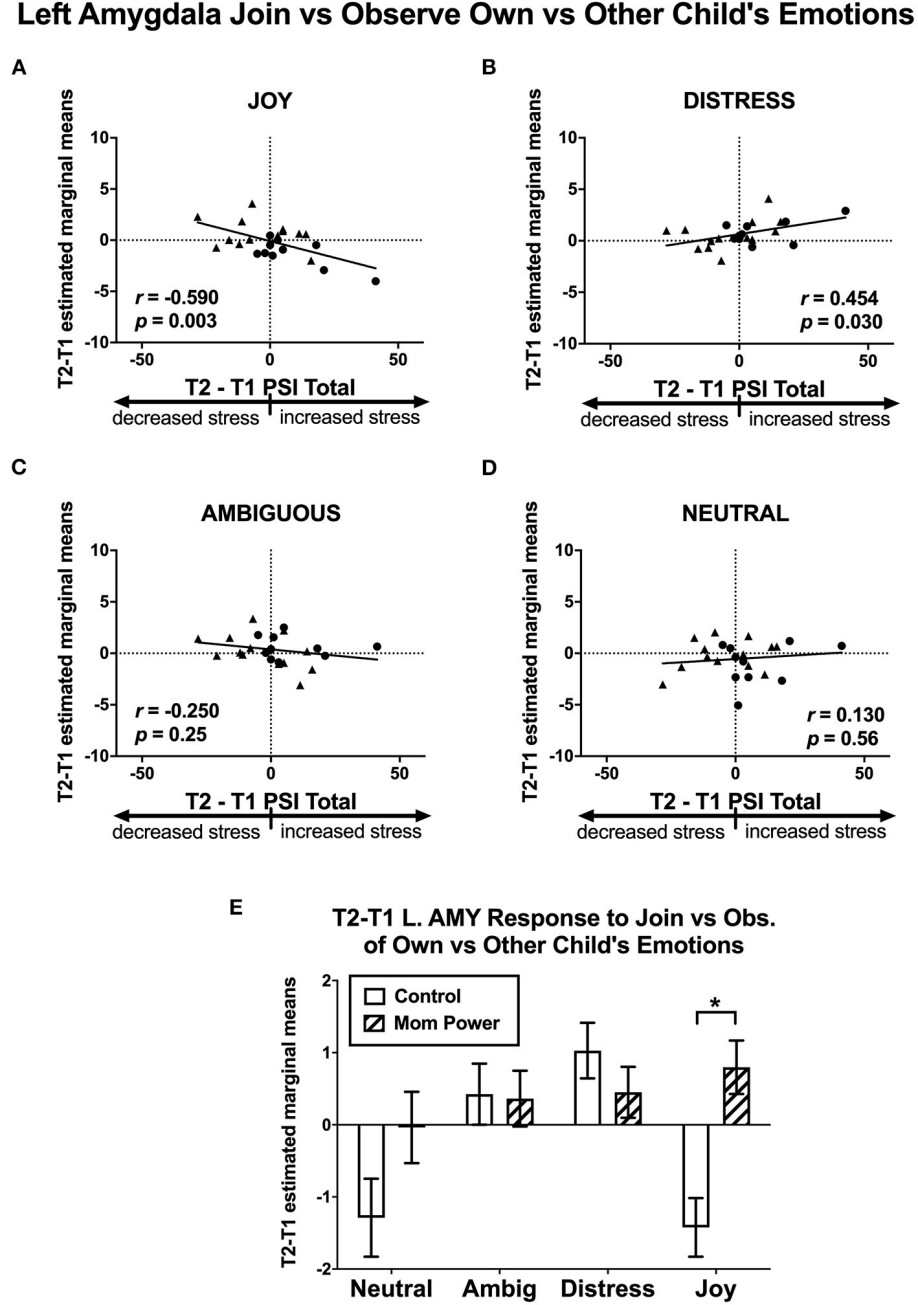
Scatter plots of Study 2 T2-T1 changes in PSI (x-axis) and T2-T1 differential responses in the left amygdala (y-axis) in the contrasts of MMR(joy) **(A)**, MMR(dis) **(B)**, MMR(amb) **(C)**, and MMR(neu) **(D)**. The T2-T1 left amygdala MMR(all) responses were increased in MP but decreased in Control group **(E)**. *p < 0.05.

Thirdly, we identified candidates of potential mediators by regressing the T1-to-T2 changes in the MMR(j-d) against dPSI, controlling for the baseline PSI at T1. We found that the T1-to-T2 reduction of parenting stress was associated with the T1-to-T2 MMR(j-d) increases in the left amygdala ([−22, 6, −18], 122 voxels, *Z* = 3.62, *p* = 0.014 s.v.c., Figure 9A), right NAc ([8, 4, −8], 30 voxels, *Z* = 3.22, *p* = 0.049 s.v.c., Figure 9B), and PAG ([−8, −32, −16], 181 voxels, *Z* = 4.34, *p* = 0.001 s.v.c., Figure 9C). Furthermore, when examining each type of expression separately (Figures 10A–D), the T1-to-T2 reduction in parenting stress was associated with the T1-to-T2 increases in the differential responses of MMR(joy) in the left amygdala (Figure 10A) and right NAc (Supplementary Figure 8A) and the T1-to-T2 decreases of MMR(joy) in the PAG (Supplementary Figure 9A). Conversely, the T1-to-T2 reduction in parenting stress was associated with the T1-to-T2 decreases in the differential responses of MMR(dis) in the left amygdala (Figure 10B) and the T1-to-T2 increases of MMR(dis) in the PAG (Supplementary Figure 9B).

Among these regions (the left amygdala, right NAc, and PAG), the MP vs. Control group difference in the MMR(j-d) was significant only in the left amygdala [*F*_(1, 21)_ = 11.51, *MS*_*error*_ = 5.79, *p* = 0.003, Figure 9D], which was primarily driven by the MP vs. Control group difference in the left amygdala's differential responses of MMR(joy) (Figure 10E). Thus, we identified the MMR(j-d) in the left amygdala as the third potential mediator, denoted as *M*_3_.

**Figure 11:**
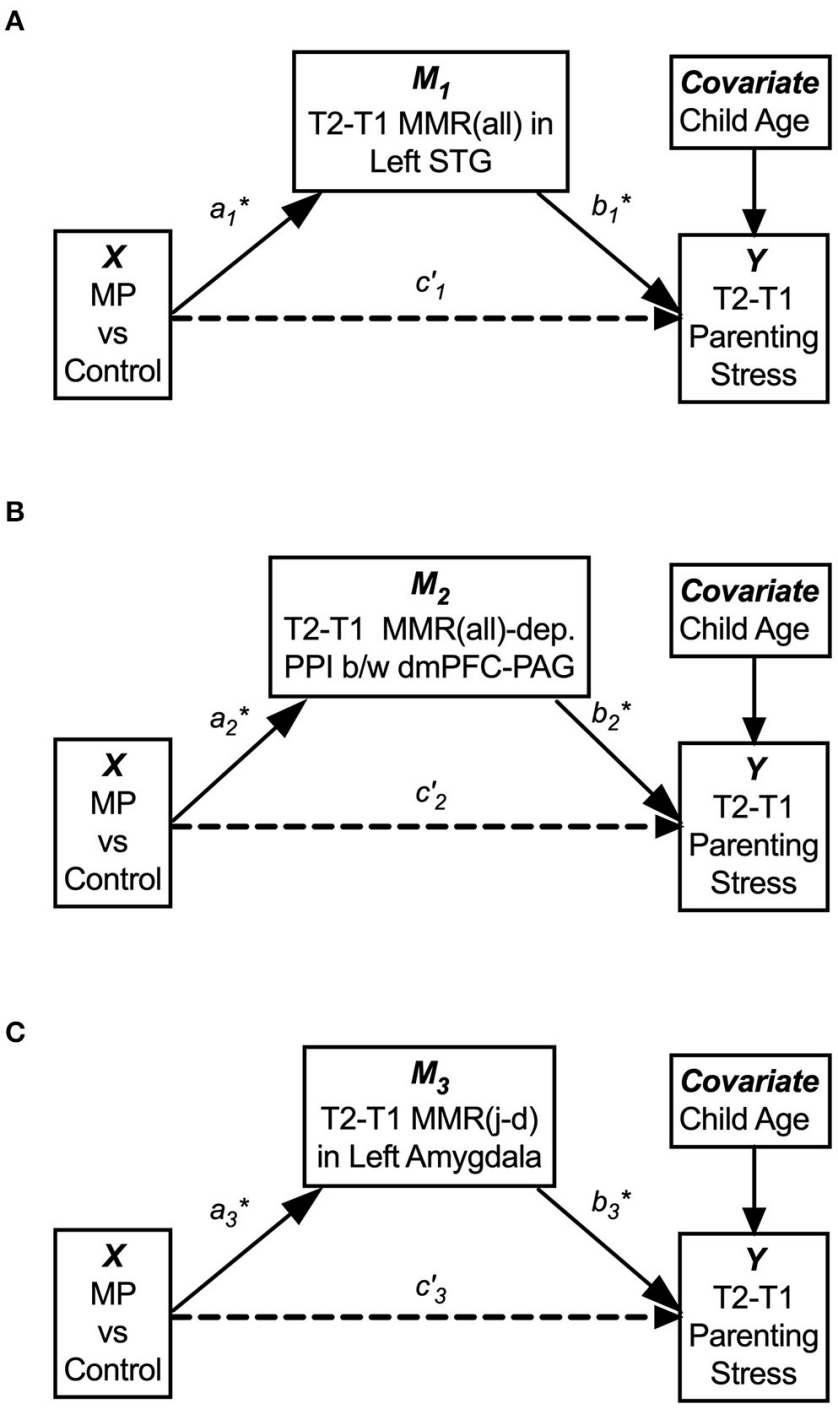
The single-mediator model for each of the three mediators: M_1_ = T2–T1 differences in the MMR(all) in the left STG **(A)**, M_2_ = T2–T1 differences in MMR(all)-dependent PPI between dmPFC and PAG, **(B)** and M_3_ = T2–T1 differences in the MMR(j-d) in the left amygdala showed that each mediator significantly mediated the MP effects on reducing parenting stress from T1 to T2 **(C)**. The age of Own Child was used as a covariate in all mediation models. See Table 3 for the statistical results of these three single-mediator models.

By running mediation analysis separately for the three potential mediators, *M*_1_ [the MMR(all) in the left STG], *M*_2_ [the MMR(all)-dependent PPI between the dmPFC-PAG], and *M*_3_ [the MMR(j-d) in the left amygdala], we found that each of them potentially mediated the indirect effect of MP treatment (*X*) on dPSI (*Y*), with <5% chance that the null hypothesis *H*_0_: *a*_*i*_*b*_*i*_ = 0 is true, as their 95% confidence interval (*c.i*.) did not cover zero. See Figure 11 and Table 3 for the statistical results for these three single-mediator models.

**Table 3:** Summary of separate single-mediator models.

**Separate models**	**Path-*a_i_***			**Path-*b_i_***			**Path-*c'_i_***			**Indirect effect (Path-*a_i_b_i_*)**			
	**Coef**.	**s.e**.	***p***	**Coef**.	**s.e**.	***p***	**Coef**.	**s.e**.	***p***	**Effect**	**s.e**.	**LLCI**	**ULCI**
M_1_	0.431	0.157	0.013	−23.669	7.583	0.006	−2.326	6.247	0.714	−10.192*	5.045	−22.688	−2.172
M_2_	−0.171	0.046	0.0013	82.432	25.869	0.005	1.555	6.890	0.824	−14.074*	7.686	−32.478	−2.789
M_3_	3.452	1.063	0.004	−3.730	14.080	0.003	0.359	6.345	0.956	−12.877*	6.041	−27.433	−3.233

*M_1_: T2-T1 MMR(all) in the left STG*.

*M_2_: T2-T1 MMR(all)-dependent PPI between dmPFC-PAG*.

*M_3_: T2-T1 MMR(j-d) in the left amygdala*.

* *95% confidence interval did not cover zero*.

*LLCI/ULCI: Lower/upper limit of 95% confidence interval*.

When these three mediators were included simultaneously in a three-mediator model, denoted as $M_{1}^{\prime }$, $M_{2}^{\prime }$, and $M_{3}^{\prime }$, respectively, we found that the relative indirect effect of $M_{1}^{\prime }$ [the MMR(all) in the left STG] was potentially stronger than those of $M_{2}^{\prime }$ [the MMR(all)-dependent PPI between the dmPFC-PAG] and $M_{3}^{\prime }$ [the MMR(j-d) in the left amygdala]. See Supplementary Figure 10 and Supplementary Table 3 for the statistical results of the three-mediator model.

## Discussion

In this translational study, at an empirical level of analysis, we employed the Child Face Mirror Task (CFMT) to examine brain mechanisms underlying maternal intersubjectivity problems, with specific focus on two problem domains of “over-mentalizing” and “under-coupling,” and to showcase the MP interventions effects on reversing these “over-mentalizing” and “under-coupling” problems, which ultimately links to reductions in parenting stress. In addition, at an abstract level of analysis to be presented at the end of this paper (section Abstract Level of Analysis—Toward an Overarching Framework for Research on Intersubjectivity), we address the theoretical relationship between the “over-mentalizing” and “under-coupling” problems and parenting stress, using the dyadic active inference framework. By combining both empirical and theoretical levels of analysis, we hope to have provided an enriched conceptual model for future research on intersubjectivity and mother-child interaction. We hereby summarize the results in support of the predictions first in section A Summary in Support of the Predictions and then discuss the results in more details in sections Neural Bases of Empathic Mirroring, The Roles of Dorsomedial Prefrontal Cortex (dmPFC), The Roles of Amygdala, The Roles of Nucleus Accumbens (NAc) and Periaquaductal Gray (PAG), The Roles of Superior Temporal Gyrus (STG), and The Roles of Prefrontal Cortex.

### A Summary in Support of the Predictions

We hypothesized that MP can reduce parenting stress by improving the mothers' working models of the child, which in turn improve maternal empathic mirroring of the child's joyful expressions (reversal of “under-coupling”) and prevent mothers' defensive reactions from shaping their mental representation of their child (reversal of “over-mentalizing”) during empathic mirroring. The hypothesis was supported by the results in the following group-by-time interaction effects during the CFMT: We found that MP (vs. Control), from T1 to T2, (1) reduced parenting stress (Supplementary Figure 2), (2) decreased the dmPFC (in the *mentalizing system*) activities during own-child-specific voluntary responding (React to Own vs. Other's Child), suggesting that MP rectified the “over-mentalizing” problem (Figure 4), (3) increased MMR(all) (own-child-specific empathic mirroring) in the *mirroring system* (Figure 6), and (4) the amygdala's MMR(j-d), i.e., the sensitivity to the prediction errors (Figure 5), suggesting that MP rectified the “under-coupling” problem. The results also supported our predictions that, from T1 to T2, MP (vs. Control) (5) reversed the stress-potentiated “under-coupling” problem, suggested by the association between the increasing sensitivity to *signed prediction errors* in the amygdala's MMR(j-d) and the decreasing parenting stress index (PSI) (Figure 9) and (6) reversed the stress-potentiated over-mentalizing problem, suggested by the association between the decreasing MMR(all)-dependent dmPFC-PAG functional connectivity and the decreasing PSI (Figure 8). We also identified three potential brain mediators of the MP treatment effects on reducing parenting stress: (1) the T1-to-T2 increases in the MMR(all) of the left STG, (2) the T1-to-T2 decreases in the MMR(all)-dependent psychological-physiological interaction (PPI) between the dmPFC and PAG, and (3) that the T1-to-T2 increases in the MMR(j-d) of the left amygdala. The results of these potential mediators will be discussed later.

### Neural Bases of Empathic Mirroring

In Study 1, the results in the contrast between strong coupling (Join) and weak coupling (Observe) conditions is highly consistent with the predictions deduced from our novel dyadic active inference framework. Specifically, the Join > Observe contrast primarily activated the *mirroring system*, along with the *salience network*, including the SMA, pericentral cortex, inferior parietal lobule (IPL), insula, thalamus, striatum, and left amygdala. Conversely, the Join > Observe primarily deactivated the *mentalizing system*, including the dmPFC, precuneus/posterior middle cingulate cortex, parahippocampal gyrus/hippocampus, and OFC, along with the visual cortex. Furthermore, some of these Join vs. Observe results overlapped with the Join vs. React results. Specifically, the strong coupling condition of Join (vs. both React and Observe) activated the bilateral pericentral cortex and left IPL, but deactivated the dmPFC, primary and secondary visual cortices, and right hippocampus.

### The Roles of Dorsomedial Prefrontal Cortex (dmPFC)

According to the affect-object active inference model (40), the dmPFC may mediate the mentalization of others (as a distal-object sketchpad to hold affective active inference of a counterpart), and it has been found that the dmPFC mediated mentalization based on a self-centered, rather than other-centered, perspective (46). The down-regulation of the dmPFC responses during the strong coupling condition (Join) in the healthy mothers in Study 1 probably help preserve their maternal intersubjectivity by preventing the over-mentalizing problem, which may manifest as perspective mistaking that can happen when one overly relies on preconceived beliefs (88). In short, it is probably necessary to suspend (temporarily down-regulate) the prior-driven dmPFC to avoid the over-mentalizing problem and achieve a higher level of intersubjectivity in a strong coupling condition.

The dmPFC has been known to be sensitive to repeated stress (89, 90) and postpartum depression (91). In accord, we previously found that, when listening to own baby's crying, the maternal dmPFC response (92) and its functional connectivity with anxiety-dependent extended amygdala (93) increased with maternal stress-related symptoms. The present study suggested a new insight into the roles of dmPFC in stress resilience, i.e., the dmPFC mediated maternal preconceived beliefs of the child as part of the *mentalizing system*, which should be temporarily suspended when the mothers relied on the *mirroring system* to empathically mirror the child. Moreover, MP enhanced the maternal capacity to down-regulate the dmPFC voluntarily while responding to own child and probably reduced parenting stress by diminishing the influences of PAG-dependent defensive/aggressive motivation signals on the dmPFC-dependent (preconceived) representation of the child. In other words, interpersonal stress can be reduced if defensive signals from the PAG are prevented from influencing the dmPFC, otherwise it would cause the defensive over-mentalizing problem that tends to increase stress.

### The Roles of Amygdala

With regard to the amygdala, we found that, in Study 1, (a) the left amygdala was activated in Join vs. Observe and (b) the left amygdala was sensitive to MMR(j-d); in Study 2, (c) from T1 to T2, the left amygdala's MMR(joy) (Join vs. Observe of Own vs. Other Child's Joyful expression) increased in MP, relative to Control, (d) from T1 to T2, the bilateral amygdala (and other regions in the maternal motivation and mirroring component) increased their MMR(all) responses in MP, relative to Control, and (e) T1-to-T2 increases in the left amygdala MMR(j-d) responses mediated the MP effects on reducing parenting stress.

The constellation of amygdala-related results provided more nuanced understanding of the amygdala's role in maternal behaviors, in accordance with the literature documenting the roles of amygdala in parental synchrony in interactions with the infant (94), empathy for the own child (71), positive feelings and attachment to the infant (95), and autobiographical recall of positive and negative emotion cues (96).

### The Roles of Nucleus Accumbens (NAc) and Periaquaductal Gray (PAG)

Consistent with the roles of NAc and PAG in maternal affiliative and defensive motivations, respectively (52, 53, 78) and their roles in *signed prediction errors* of reward (55, 56) and pain (59), respectively, we found that these two regions were related to the T1-to-T2 changes in parenting stress in opposite directions. While the T1-to-T2 changes in parenting stress were negatively associated with the NAc's MMR(j-d) and MMR(all)-dependent PPI with the dmPFC, it was positively associated with the PAG's. Consistent with the affect-object active inference model (40), these results highlights the role of dmPFC as a distal-object sketchpad in representing the child and the “coloring” of the representation with affiliative and defensive affective potentials, forming “affect-objects,” by its connectivity with NAc and PAG (59), respectively. So, this suggests that the role of affect-object generation during empathic mirroring in parenting stress, i.e., mirroring the child with affiliative or defensive affective potentials can decrease or increase parenting stress, respectively.

### The Roles of Superior Temporal Gyrus (STG)

We also found that the T1-to-T2 reduction in parenting stress was associated with the concomitant increases in the MMR(all) in the left STG, right STG/Insula, cerebellum, and hypothalamus. Interestingly, the first three regions were related to music-entrained movement coherences in professional dancers (97), suggesting that increasing coherence in empathic mirroring may be related to parenting stress reduction. In a cross-culture study, these brain regions were commonly activated when mothers from different cultures listened to their own baby's cry (98). Consistent with the result that the left STG mediated the MP effects on reducing parenting stress in the present study, we have reported that the T1-to-T2 parenting stress reduction was associated with the concomitant increases in the functional connectivity between the left STG and amygdala, when the mothers responded to own baby's crying (29). Maternal STG responses to own vs. other's infant cry were associated with child-oriented caring thoughts and indirectly with infant development (99). Taken together, these results implicated that parenting stress reduction may depend on increasing the coherence in maternal empathic mirroring of the child, which is potentially mediated by the amygdala-STG neurocircuits, as part of the mirroring system.

### The Roles of Prefrontal Cortex

We also found that the left prefrontal cortex was activated in Join vs. Rest (Figure 2C), in accord with a recent hyper-scanning study that reported increasing maternal parenting stress was also associated with the differences between mother and child's left prefrontal cortex responses when the dyads watched videos together (100). Considering that the left prefrontal cortex is part of the mirroring system (32), these results suggested that parenting stress may influence the mother-child coupling via the left prefrontal cortex.

### Limitations

Several limitations of the present study should be noted. First, although Study 1 established the intended effects of CFMT with whole brain correction in a relatively large sample (*n* = 45), the sample sizes of MP and Control groups in Study 2 were modest and thus the results should be considered preliminary and warrant future study. Second, there was heterogeneity in medication use in Study 2. Nevertheless, this heterogeneity would cause little confounding because not only it was partially controlled in the repeated measurement effects based on each participant's own baseline, but also the medicated participants were in minority and evenly distributed between the groups. As reported in Supplementary Materials, removing all medicated participants in statistical analysis did not qualitatively alter the results reported above. Third, we did not incorporate measurements that are directly linked to the “over-mentalizing” and “under-coupling” problems in the parenting context. Nevertheless, the effectiveness of MP's improvement on the symptoms of “over-mentalizing” and “under-coupling” has been documented (28) and thus the reported Time-by-Group interaction results should be closely related to the correction of “over-mentalizing” and “under-coupling” problems. We will examine the associations between the neuroimaging data and these variables in the future.

## Abstract Level of Analysis—Toward an Overarching Framework For Research on Intersubjectivity

In this section, we describe a dyadic active inference framework, at an abstract level of analysis, to address theoretical relationships between the impairment of intersubjectivity and parenting stress we well as to clarify the relationships among the dyadic framework, the MP intervention, and the brain systems. First, we introduce a single-agent active inference framework, namely Free Energy Principle (FEP) (67, 68, 101). Second, we propose a novel *dyadic active inference framework* to account for the link between intersubjectivity and stress resilience in a two-agent system (mother and child dyad). Third, we explain the links between the impairment of intersubjectivity and parenting stress based on the dyadic framework. Fourth, we interpret the MP intervention in light of the dyadic framework. Last, we map brain systems to the components of the active inference framework.

### Single-Agent Active Inference Framework

Bayesian active inference (also known as predictive coding) is a computationally powerful framework, as its variants not only can account for perception, cognition, emotions, and consciousness in humans and animals (35, 40, 67, 69, 102–108), but also biologic evolution (109) and even artificial intelligence (110, 111).

According to FEP (67, 68), an agent's predictive-coding engine can be heuristically modeled in a hierarchical network, which contains four nodes (**E**, **S**, **A**, and **M**) in three levels: **E** is Event from environments at the bottom, **S** is Sensation and **A** is Action at the intermediate, and **M** is the internal prior Model at the top level (Figure 12A). When an event **E** causes **S** to generate afferent data, **S** causes **M** to predict what the event *means* based on stored prior causal models, and **M** in turn causes **A** to respond to the event, and then the differences between **S** (the afferent data) and **A** (the efferent prediction) are computed, serving as prediction errors in feedback to update the priors in **M**. Because there is no direct contact between **M** and **E** nodes, the engine depends on the prediction errors resulting from the interactions between the agent's **S and A** to infer the events in **E**. The interactions between an agent's **S** and **A** and events (**E**) update the internal model **M** iteratively, until the prediction errors are minimized, and **M** is thus optimized.

**Figure 12:**
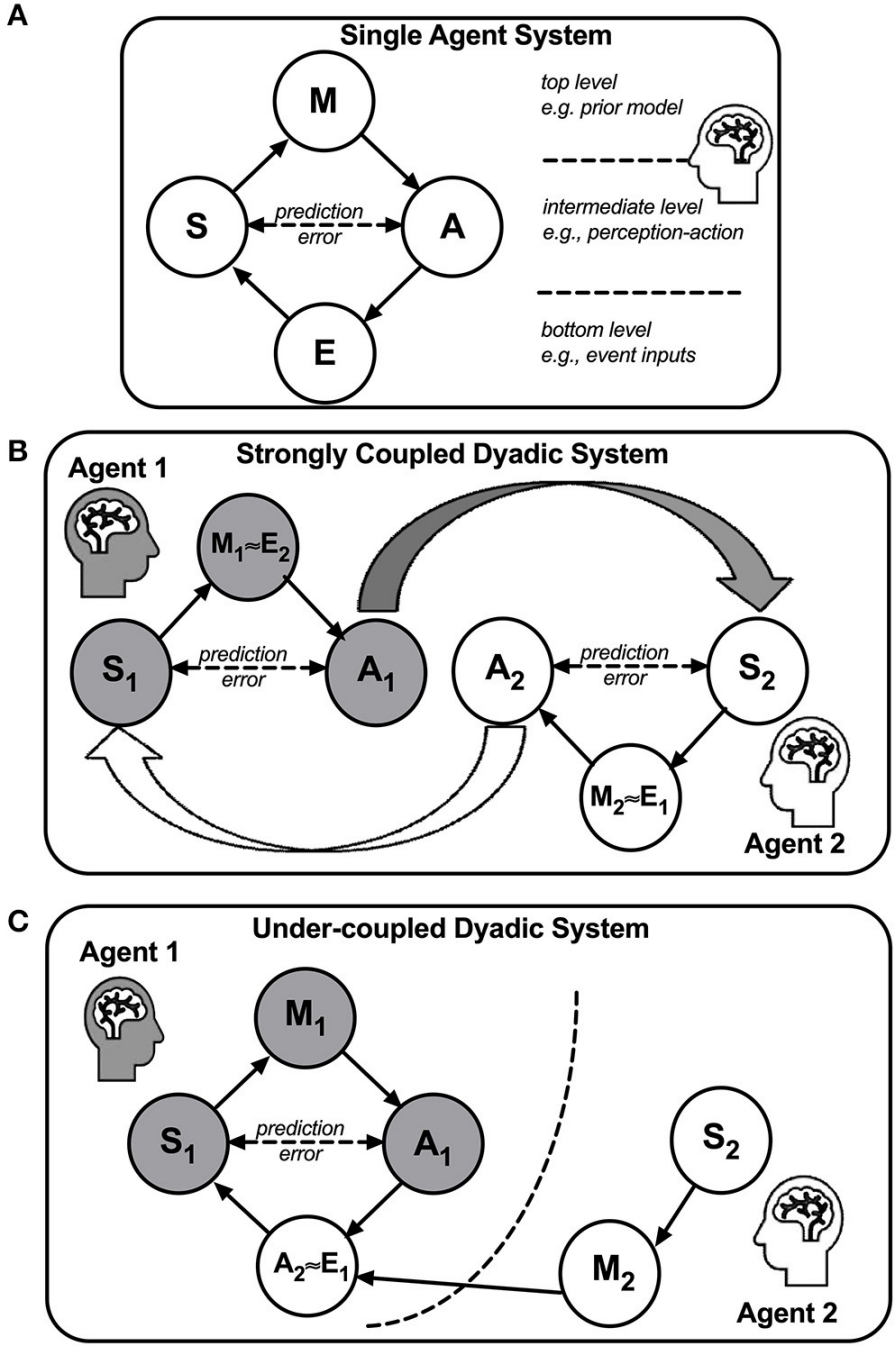
A Bayesian active inference framework for a single-agent system **(A)**, a strongly coupled dyadic system **(B)**, and an under-coupled dyadic system **(C)**. In **(A)**, an agent and its environments form a single-agent system, depicted as a four-node hierarchical network. E, a node representing events from environments at the bottom level; S, a node representing the agent's sensation; A, a node representing the agent's action; M, a node representing the agent's internal model. The S and A nodes are positioned at the intermediate level and the M node is positioned at the top level. The *prediction error*, defined as the difference between the data in the S node and the prediction in the A node, is bounded by free energy. When the free energy is minimized by the M node, the agent can reliably predict the environments, and thus the adaptation of the agent to the environments is optimized. In **(B)**, a strong coupling between two agents is formed when Agents 1 and 2 are coupled by their S's and A's nodes, wherein A_1_ causes S_2_ and A_2_ causes S_1_. Due to the coupling, each agent's prediction errors are also coupled and thus the adaptation is optimized when the collective free energy is minimized. In an optimal state, M_1_ and M_2_ will be highly consistent with one another, indicating a high level of intersubjectivity. In **(C)**, under-coupling ensues when Agent 1 discards Agent 2's M_2_ and S_2_ and instead only focuses on Agent 2's behaviors A_2_ in relation to Agent 1's S_1_ and A_1_. Due to the under-coupling, Agent 1 tends to misattribute the causes of Agent 2's behaviors.

### Dyadic Active Inference Framework

The notion of human as a social active inference engine has emerged in the recent literature (35, 40, 106, 108). As social interactions lie at the core of intersubjectivity, single-agent active inference framework is simply inadequate to account for intersubjectivity. Thus, we propose a novel *dyadic* active inference framework to model intersubjectivity (Figure 12B). In this dyadic framework, in a two-agent coupled system wherein Agent 1 (say, Mom) and Agent 2 (say, Son) are strongly coupled such that one agent's action (**A**) *predominantly* causes the other's sensation (**S**) and *vice versa*, i.e., **A**_***Mom***_ **≈** **S**_***Son***_ and **A**_***Son***_ **≈** **S**_***Mom***_, each agent's internal model (**M**) will serve as the other's events **E**, i.e., **M**_***Son***_ **≈** **E**_***Mom***_ and **M**_***Mom***_ **≈** **E**_***Son***_. When Mom's internal model (her working model of the child) approximate Son's (his working model of the mother), **M**_**Mom**_ **≈** **M**_***Son***_, she achieves intersubjectivity and minimizes her prediction errors in the dyadic system.

One question arises that if **M**_**Mom**_ **≈** **M**_***Son***_, then the mother's working model (**M**_***Mom***_) will be as helpless as the son's (**M**_***Son***_) when the son struggles in distress. This would not be the case if the mother would possess more knowledge or wisdom, i.e., if her working model could access more repertoires or strategies that the son's does not have. *It is important to note that the presence or absence of* **M**_***Mom***_ **≈** **M**_***Son***_ *as a state is transactional, not permanent*. Therefore, after the mother achieves the state of **M**_**Mom**_ **≈** **M**_***Son***_, she can access additional resources, repertoires, or strategies and then teach the son to expand his working model to solve his issue at hand. Conversely, without first achieving the state of **M**_**Mom**_ **≈** **M**_***Son***_, the mother may fail to address what the son needs or to teach him any new strategies effectively because she may have misunderstood what the son actually needs in that current moment.

### Linking Intersubjectivity and Stress in the Dyadic Framework: Three Propositions

Our dyadic framework can make sense of why *intersubjectivity* can automatically minimize stress in a two-agent coupled system. Recently, stress has been re-defined as uncontrollable prediction errors (excessive free energy) that threatens the agent as a Bayesian active inference engine (69). Thus, the minimization of prediction errors is equivalent to the minimization of stress. When two or more agents are coupled as a relational whole, if one agent merely projects one's own beliefs about another agent's perception, action, and intention—without relying on data from ongoing dyadic interactions—the prediction errors will tend to increase, as exemplified in *perspective mistaking* (88).

How can imitation facilitate intersubjectivity? In the two-agent system, when the mother imitates the child's action (e.g., smile), their actions are similar and their perceptions are also similar (e.g., joy). By virtue of such reciprocal similarity, the dyad can better predict each other's covert working models underlying their actions and perceptions. Thus, imitation can reduce prediction errors in predicting each other's actions and feelings, which may in turn increase the similarity between their covert working models underlying those actions and feelings, thereby facilitating intersubjectivity. However, when the mother's preconceived working model of the child is fixated in negative mood under stress (excessive prediction errors), her capacity to utilize prediction errors to update her working model of the child, which would have helped her better imitate the child's positive affective expressions, is compromised.

We hereby link intersubjectivity and stress in terms of the dyadic active inference framework in three inter-related propositions, namely *dyadic symbiosis, under-coupling*, and *over-mentalizing*, as follows:

1. *A strongly-coupled dyadic system is symbiotic*: When a dyad's **S's** and **A's** are strongly coupled (**A**_***Mom***_ **≈** **S**_***Son***_ and **A**_***Son***_ **≈** **S**_***Mom***_), they function in *symbiosi*s, in which the prediction errors are minimized collectively *if* , and *only if* , the prediction error in one agent is minimized without increasing the other's. In such symbiosis, Mom can achieve intersubjectivity (**M**_**Mom**_ **≈** **M**_**Son**_) by minimizing her prediction errors through communicative interactions with Son. When an agent supports self and other's intentions symbiotically, the agent is considered to be maintaining a stance of intersubjective benevolence.
2. *Under-coupling increases prediction errors*: As depicted in Figure 12C, when Agent 1's **S**_**1**_ and **A**_**1**_ engage Agent 2's **A**_**2**_ only, Agent 1 will ignore Agent 2's **M**_**2**_ and **S**_**2**_ and thus Agent 1 may fail to achieve intersubjectivity and find it difficult to reduce stress in either agent. For example, when Mom neglects how her harsh reactions (**A**_***Mom***_) make Son feel (**S**_***Son***_) and only focuses on how to change Son's actions (**A**_***Son***_), Mom would fail to recognize Son's internal model (**M**_***Son***_) and therefore Mom's prediction errors about Son's internal model and behaviors would increase. Being ignored or rejected, Son's stress (excessive free energy) would increase, which increases Mom's stress in return.
3. *Stress-potentiated over-mentalizing perpetuates intersubjectivity impairments*: When dyadic stress increases, Agent 1 may become defensive against Agent 2, as if Agent 2 were an enemy, and therefore misattribute Agent 2's disagreeing behaviors to malice or character flaw, i.e., over-mentalizing. For example, Mom may over-mentalize Son's behaviors as “he means to upset me” or “he is mean.” When Mom's over-mentalizing explains away Son's actual internal model, she will not even recognize her own ignorance of Son's feelings (**S**_***Son***_) and psychological needs (**M**_***Son***_). Thus, when stress potentiates Mom's over-mentalizing, Son's disagreeing behaviors would only confirm Mom's prior models of stereotypical biases against him, and under this condition, the problems of over-mentalizing, under-coupling, and intersubjectivity impairment will continue in a vicious cycle.

### Interpreting MP in Light of the Dyadic Active Inference Framework

We hereby interpret MP intervention in light of the dyadic framework.

1. MP cultivates mothers' knowledge and skills to address a child's psychological needs to promote maternal intersubjectivity through (a) didactic teachings of attachment theory and developmental principles and (b) facilitated mother-child interactions.
2. MP rectifies under-coupling problems by increasing maternal awareness of how a child's overt behaviors (**A**_***Son***_) may communicate underlying (covert) feelings (**S**_***Son***_) and psychological needs (**M**_***Son***_).
3. MP curbs stress-potentiated over-mentalizing problems via enhancing maternal distress tolerance and non-judgmental stance through teaching mindfulness-based stress regulation skills.

### Mapping Brain Systems to the Active Inference Framework

The three systems in the social brain, i.e., the *mirroring system, mentalizing system*, and *salience network* can be mapped to three components of the active inference framework. As depicted in Figure 1A, social cognition can be modeled as a hierarchical network of active inference engines, which encompasses: (1) an intermediate level involving *mirroring system* as a bottom-up component for automatic perception-action coupling, (2) the *salience network* as a feedback component mediating the surprise (i.e., socially salient prediction errors) detected in the intermediate level, and (3) a top level involving *mentalizing system* as a top-down component for affective and relational model to simulate relationships between self and others.

The functional distinction between the *mirroring system* and *mentalizing system* has gained empirical supports (112). As a bottom-up process, mirroring can be performed spontaneously without activating higher-order representations (16). In contrast, as a top-down process, while the *mentalizing system* can be activated by the theory-of-mind tasks (45), retrospective remembering and proactive imagining of episodic memory (113), and belief-based social attribution (46), it can also be spontaneously active without any inputs or task demands, as part of the default-mode network (61). The roles of the salience network in (a) conflict monitoring (49), (b) switching dynamic oscillations between the frontoparietal network (overlapping with the *mirroring system*) and the default-mode network (overlapping with the *mentalizing system*) during resting (66), and (c) representing *signed prediction errors* of reward (55, 56) and punishment (58, 59) are consistent with its potential role in the prediction errors as a feedback from the *mirroring system* to the *mentalizing system*.

### Conclusion

This study advances the science of intersubjectivity and stress resilience on multiple levels. On a theory-generating level, we utilized a promising dyadic active inference framework and offered theoretical relationships between the “over-mentalizing” and “under-coupling” intersubjectivity problems and parenting stress. Further, on an empirical level, we proposed a novel fMRI task to identify neurocircuitry underlying intersubjectivity and potential mediators of the intersubjectivity-oriented intervention (Mom Power). Combined with the within-subject changes afforded by MP intervention, our results point to a two-pronged and potentially generalizable principle, i.e., stress resilience depends on not only mitigating stress-potentiated under-coupling and over-mentalizing problems, but also enhancing a stance of intersubjective benevolence in mirroring others' feelings and serving their well-being in dyadic symbiosis.

## Data Availability Statement

The raw data supporting the conclusions of this article will be made available by the authors, without undue reservation.

## Ethics Statement

The studies involving human participants were reviewed and approved by Institutional Review Board at University of Michigan, Ann Arbor, MI. The patients/participants provided their written informed consent to participate in this study.

## Author Contributions

SH was the principal developer of the theoretical framework and hypotheses and writer of the manuscript. He designed and programmed the fMRI task and conducted the data analysis of the study. He created the figures in the study. MM was one of the developers of Mom Power and contributed to study design and implementation in the community setting, including grant support. She contributed to manuscript development conceptually, and wrote together with SH the first draft, as well as provided ongoing major manuscript edits. KR was also one of the developers of Mom Power. She co-designed and oversaw Study 2 and provided supervision for study clinicians. She contributed to study design and implementation in the community setting. She contributed by writing sections of the manuscript and through ongoing edits. DM contributed to this manuscript with conceptual/content expertise on relationship-based parenting interventions, through contributing to drafting sections of the manuscript, and through providing ongoing edits and feedback. YN collaborated with SH in developing and refining an intersubjectivity-based theoretical framework for understanding how two agents interact with and understand each other. He also provided suggestions for data analysis and ongoing edits in the manuscript. JS was the senior investigator in the study. He co-designed the fMRI task and provided overall supervision on the fMRI studies. He contributed by obtaining grant support for the research and providing theoretical justifications, background, and manuscript edits. All authors contributed to the article and approved the submitted version.

## Conflict of Interest

The authors declare that the research was conducted in the absence of any commercial or financial relationships that could be construed as a potential conflict of interest.

## References

[1] Williford AP Calkins SD Keane SP. Predicting change in parenting stress across early childhood: child and maternal factors. J Abnorm Child Psychol. (2007) 35:251–63. 10.1007/s10802-006-9082-3 17186365

[2] Neece CL Green SA Baker BL. Parenting stress and child behavior problems: a transactional relationship across time. Am J Intell Dev Disabil. (2012) 117:48–66. 10.1352/1944-7558-117.1.48 22264112 PMC4861150

[3] Camoirano A. Mentalizing makes parenting work: a review about parental reflective functioning and clinical interventions to improve it. Front Psychol. (2017) 8:14. 10.3389/fpsyg.2017.00014 28163690 PMC5247433

[4] Bernard K Nissim G Vaccaro S Harris JL Lindhiem O. Association between maternal depression and maternal sensitivity from birth to 12 months: a meta-analysis. Attach Hum Dev. (2018) 20:578–99. 10.1080/14616734.2018.1430839 29374991

[5] Zahavi D Overgaard S Intersubjectivity. In: Lafollette H, editor. International Encyclopedia of Ethics (2013).

[6] Preston SD Hofelich AJ The many faces of empathy: parsing empathic phenomena through a proximate, dynamic-systems view of representing the other in the self. Emotion Rev. (2012) 4:24–33. 10.1177/1754073911421378

[7] Rowe CE Macisaac DS Empathic Attunement: the “Technique” of Psychoanalytic Self Psychology. Lanham, MD: Jason Aronson: Rowman & Littlefield (2004).

[8] Fonagy P Steele H Steele M. Maternal representations of attachment during pregnancy predict the organization of infant-mother attachment at one year of age. Child Dev. (1991) 62:891–905. 10.2307/1131141 1756665

[9] Slade A. Parental reflective functioning: an introduction. Attach Hum Dev. (2005) 7:269–81. 10.1080/14616730500245906 16210239

[10] Ainsworth MS Blehar MC Waters E Wall S Patterns of Attachment: A Psychological Study of the Strange Situation. Oxford: Erlbaum (1978).

[11] Bernard K Meade EB Dozier M. Parental synchrony and nurturance as targets in an attachment based intervention: building upon Mary Ainsworth's insights about mother–infant interaction. Attach Hum Dev. (2013) 15:507–23. 10.1080/14616734.2013.820920 24299132 PMC3855268

[12] Shai D Belsky J When words just won't do: introducing parental embodied mentalizing. Child Dev Perspect. (2011) 5:173–80. 10.1111/j.1750-8606.2011.00181.x

[13] Meltzoff AN Moore MK Imitation of facial and manual gestures by human neonates. Science. (1977) 198:75–8. 10.1126/science.198.4312.75 17741897

[14] Trevarthen C Aitken KJ. Infant intersubjectivity: research, theory, and clinical applications. J Child Psychol Psychiatry. (2001) 42:3–48. 10.1111/1469-7610.00701 11205623

[15] Kim S Fonagy P Allen J Martinez S Iyengar U Strathearn L. Mothers who are securely attached in pregnancy show more attuned infant mirroring 7 months postpartum. Infant Behav Dev. (2014) 37:491–504. 10.1016/j.infbeh.2014.06.002 25020112 PMC4301602

[16] Carr EW Winkielman P. When mirroring is both simple and “smart”: how mimicry can be embodied, adaptive, and non-representational. Front Hum Neurosci. (2014) 8:505. 10.3389/fnhum.2014.00505 25071532 PMC4095561

[17] Kohut H. Introspection, empathy, and the semi-circle of mental-health. Int J Psycho Anal. (1982) 63:395–407. 7152804

[18] Konrath SH Obrien EH Hsing C. Changes in dispositional empathy in american college students over time: a meta-analysis. Person Soc Psychol Rev. (2010) 15:180–98. 10.1177/1088868310377395 20688954

[19] Leerkes EM. Maternal sensitivity during distressing tasks: a unique predictor of attachment security. Infant Behav Dev. (2011) 34:443–6. 10.1016/j.infbeh.2011.04.006 21616538 PMC3134119

[20] Fuchs T. The intersubjectivity of delusions. World Psychiatry. (2015) 14:178–9. 10.1002/wps.20209 26043331 PMC4471970

[21] Dayton CJ Huth-Bocks AC Busuito A. The influence of interpersonal aggression on maternal perceptions of infant emotions: associations with early parenting quality. Emotion. (2016) 16:436–48. 10.1037/emo0000114 26709859

[22] Shai D Dollberg D Szepsenwol O. The importance of parental verbal and embodied mentalizing in shaping parental experiences of stress and coparenting. Infant Behav Dev. (2017) 49:87–96. 10.1016/j.infbeh.2017.08.003 28818676

[23] Schmidt D Seehagen S Hirschfeld G Vocks S Schneider S Teismann T Repetitive negative thinking and impaired mother–infant bonding: a longitudinal study. Cogn Ther Res. (2017) 41:498–507. 10.1007/s10608-016-9823-8

[24] Rosenblum KL Mcdonough SC Sameroff AJ Muzik M. Reflection in thought and action: maternal parenting reflectivity predicts mind-minded comments and interactive behavior. Infant Mental Health J. (2008) 29:362–76. 10.1002/imhj.20184 28636158

[25] Muzik M Rosenblum KL Alfafara EA Schuster MM Miller NM Waddell RM. Mom Power: preliminary outcomes of a group intervention to improve mental health and parenting among high-risk mothers. Arch Womens Ment Health. (2015) 18:507–21. 10.1007/s00737-014-0490-z 25577336 PMC4439267

[26] Muzik M Rosenblum KL Schuster MM Kohler ES Alfafara EA Miller NM A mental health and parenting intervention for adolescent and young adult mothers and their infants. J Depress Anxiety. (2016) 5:233–9. 10.4172/2167-1044.1000233

[27] Rosenblum KL Muzik M Morelen DM Alfafara EA Miller NM Waddell RM. A community-based randomized controlled trial of mom power parenting intervention for mothers with interpersonal trauma histories and their young children. Arch Womens Ment Health. (2017) 20:673–86. 10.1007/s00737-017-0734-9 28647759 PMC5709180

[28] Rosenblum KL Lawler J Alfafara E Miller N Schuster M Muzik M. Improving maternal representations in high-risk mothers: a randomized, controlled trial of the mom power parenting intervention. Child Psychiatry Hum Dev. (2018) 49:372–84. 10.1007/s10578-017-0757-5 28936602 PMC5862741

[29] Swain JE Ho SS Rosenblum KL Morelen D Dayton CJ Muzik M. Parent–child intervention decreases stress and increases maternal brain activity and connectivity during own baby-cry: an exploratory study. Dev Psychopathol. (2017) 29:535–53. 10.1017/S0954579417000165 28401845 PMC7195811

[30] Van Overwalle F Baetens K. Understanding others' actions and goals by mirror and mentalizing systems: a meta-analysis. NeuroImage. (2009) 48:564–84. 10.1016/j.neuroimage.2009.06.009 19524046

[31] Decety J The neural pathways, development and functions of empathy. Curr Opin Behav Sci. (2015) 3:1–6. 10.1016/j.cobeha.2014.12.001

[32] Vogeley K. Two social brains: neural mechanisms of intersubjectivity. Philos Trans Roy Soc B Biol Sci. (2017) 372:245. 10.1098/rstb.2016.0245 28673921 PMC5498305

[33] Rizzolatti G Craighero L. The mirror-neuron system. Ann Rev Neurosci. (2004) 27:169–92. 10.1146/annurev.neuro.27.070203.144230 15217330

[34] Iacoboni M Molnar-Szakacs I Gallese V Buccino G Mazziotta JC Rizzolatti G. Grasping the intentions of others with one's own mirror neuron system. PLoS Biol. (2005) 3:e79. 10.1371/journal.pbio.0030079 15736981 PMC1044835

[35] Kilner JM Friston KJ Frith CD. Predictive coding: an account of the mirror neuron system. Cogn Processing. (2007) 8:159–66. 10.1007/s10339-007-0170-2 17429704 PMC2649419

[36] Cross KA Torrisi S Reynolds Losin EA Iacoboni M. Controlling automatic imitative tendencies: interactions between mirror neuron and cognitive control systems. NeuroImage. (2013) 83:493–504. 10.1016/j.neuroimage.2013.06.060 23811412 PMC4004608

[37] Hipwell AE Guo C Phillips ML Swain JE Moses-Kolko EL. Right frontoinsular cortex and subcortical activity to infant cry is associated with maternal mental state talk. J Neurosci. (2015) 35:12725–32. 10.1523/JNEUROSCI.1286-15.2015 26377462 PMC4571605

[38] Elmadih A Wan MW Downey D Elliott R Swain JE Abel KM. Natural variation in maternal sensitivity is reflected in maternal brain responses to infant stimuli. Behav Neurosci. (2016) 130:500–10. 10.1037/bne0000161 27513806

[39] Campbell MEJ Cunnington R. More than an imitation game: top-down modulation of the human mirror system. Neurosci Biobehav Rev. (2017) 75:195–202. 10.1016/j.neubiorev.2017.01.035 28153686

[40] Ho SS Nakamura Y Healing dysfunctional identity: bridging mind-body intervention to brain systems. J Behav Brain Sci. (2017) 7:137–64. 10.4236/jbbs.2017.73013

[41] Garrison KA Santoyo JF Davis JH Thornhill TAT Kerr CE Brewer JA. Effortless awareness: using real time neurofeedback to investigate correlates of posterior cingulate cortex activity in meditators' self-report. Front Hum Neurosci. (2013) 7:440. 10.3389/fnhum.2013.00440 23964222 PMC3734786

[42] Lindquist KA Barrett LF. A functional architecture of the human brain: emerging insights from the science of emotion. Trends Cogn Sci. (2012) 16:533–40. 10.1016/j.tics.2012.09.005 23036719 PMC3482298

[43] Denny BT Kober H Wager TD Ochsner KN. A meta-analysis of functional neuroimaging studies of self- and other judgments reveals a spatial gradient for mentalizing in medial prefrontal cortex. J Cogn Neurosci. (2012) 24:1742–52. 10.1162/jocn_a_00233 22452556 PMC3806720

[44] Li W Mai X Liu C. The default mode network and social understanding of others: what do brain connectivity studies tell us. Front Hum Neurosci. (2014) 8:74. 10.3389/fnhum.2014.00074 24605094 PMC3932552

[45] Molenberghs P Johnson H Henry JD Mattingley JB. Understanding the minds of others: a neuroimaging meta-analysis. Neurosci Biobehav Rev. (2016) 65:276–91. 10.1016/j.neubiorev.2016.03.020 27073047

[46] Sommer M Döhnel K Sodian B Meinhardt J Thoermer C Hajak G. Neural correlates of true and false belief reasoning. NeuroImage. (2007) 35:1378–84. 10.1016/j.neuroimage.2007.01.042 17376703

[47] Saxe R Powell LJ. It's the thought that counts: specific brain regions for one component of theory of mind. Psychol Sci. (2006) 17:692–9. 10.1111/j.1467-9280.2006.01768.x 16913952

[48] Van Overwalle F. Social cognition and the brain: a meta-analysis. Hum Brain Mapp. (2009) 30:829–58. 10.1002/hbm.20547 18381770 PMC6870808

[49] Seeley WW Menon V Schatzberg AF Keller J Glover GH Kenna H. Dissociable intrinsic connectivity networks for salience processing and executive control. J Neurosci. (2007) 27:2349–56. 10.1523/JNEUROSCI.5587-06.2007 17329432 PMC2680293

[50] Uddin LQ. Salience processing and insular cortical function and dysfunction. Nat Rev Neurosci. (2015) 16:55–61. 10.1038/nrn3857 25406711

[51] Laurita AC Hazan C Spreng RN. An attachment theoretical perspective for the neural representation of close others. Soc Cogn Affect Neurosci. (2019) 14:237–51. 10.1093/scan/nsz010 30715524 PMC6399606

[52] Numan M Woodside B. Maternity: neural mechanisms, motivational processes, and physiological adaptations. Behav Neurosci. (2010) 124:715–41. 10.1037/a0021548 21133530

[53] Swain JE Ho SS. Neuroendocrine mechanisms for parental sensitivity: overview, recent advances and future directions. Curr Opin Psychol. (2017) 15:105–10. 10.1016/j.copsyc.2017.02.027 28813249 PMC7195810

[54] Swain JE Ho SS Fox H Garry D Brummelte S. Effects of opioids on the parental brain in health and disease. Front Neuroendocrinol. (2019) 54:100766. 10.1016/j.yfrne.2019.100766 31128130 PMC8318357

[55] Dayan P Balleine BW Reward, motivation, and reinforcement learning. Neuron. (2002) 36:285–98. 10.1016/S0896-6273(02)00963-7 12383782

[56] Daw ND Gershman SJ Seymour B Dayan P Dolan RJ. Model-based influences on humans' choices and striatal prediction errors. Neuron. (2011) 69:1204–15. 10.1016/j.neuron.2011.02.027 21435563 PMC3077926

[57] Belova MA Paton JJ Morrison SE Salzman CD. Expectation modulates neural responses to pleasant and aversive stimuli in primate amygdala. Neuron. (2007) 55:970–84. 10.1016/j.neuron.2007.08.004 17880899 PMC2042139

[58] Mchugh SB Barkus C Huber A Capitão L Lima J Lowry JP. Aversive prediction error signals in the amygdala. J Neurosci. (2014) 34:9024–33. 10.1523/JNEUROSCI.4465-13.2014 24990922 PMC4078079

[59] Roy M Shohamy D Daw N Jepma M Wimmer GE Wager TD. Representation of aversive prediction errors in the human periaqueductal gray. Nat Neurosci. (2014) 17:1607–12. 10.1038/nn.3832 25282614 PMC4213247

[60] Yarkoni T Poldrack RA Nichols TE Van Essen DC Wager TD. Large-scale automated synthesis of human functional neuroimaging data. Nat Meth. (2011) 8:665–70. 10.1038/nmeth.1635 21706013 PMC3146590

[61] Raichle ME. The brain's default mode network. Ann Rev Neurosci. (2015) 38:433–47. 10.1146/annurev-neuro-071013-014030 25938726

[62] Fox KC Spreng RN Ellamil M Andrews-Hanna JR Christoff K. The wandering brain: meta-analysis of functional neuroimaging studies of mind-wandering and related spontaneous thought processes. Neuroimage. (2015) 111:611–21. 10.1016/j.neuroimage.2015.02.039 25725466

[63] Fox MD Snyder AZ Vincent JL Corbetta M Van Essen DC Raichle ME. The human brain is intrinsically organized into dynamic, anticorrelated functional networks. Proc Natl Acad Sci USA. (2005) 102:9673–8. 10.1073/pnas.0504136102 15976020 PMC1157105

[64] Cole MW Reynolds JR Power JD Repovs G Anticevic A Braver TS. Multi-task connectivity reveals flexible hubs for adaptive task control. Nat Neurosci. (2013) 16:1348–55. 10.1038/nn.3470 23892552 PMC3758404

[65] Cole EJ Barraclough NE Andrews TJ. Reduced connectivity between mentalizing and mirror systems in autism spectrum condition. Neuropsychologia. (2019) 122:88–97. 10.1016/j.neuropsychologia.2018.11.008 30468777

[66] Goulden N Khusnulina A Davis NJ Bracewell RM Bokde AL Mcnulty JP. The salience network is responsible for switching between the default mode network and the central executive network: replication from DCM. NeuroImage. (2014) 99:180–90. 10.1016/j.neuroimage.2014.05.052 24862074

[67] Friston K. The free-energy principle: a unified brain theory? Nat Rev Neurosci. (2010) 11:127–38. 10.1038/nrn2787 20068583

[68] Friston K. Life as we know it. J Roy Soc Interf. (2013) 10:475. 10.1098/rsif.2013.0475 23825119 PMC3730701

[69] Peters A Mcewen BS Friston K. Uncertainty and stress: why it causes diseases and how it is mastered by the brain. Prog Neurobiol. (2017) 156:164–88. 10.1016/j.pneurobio.2017.05.004 28576664

[70] Eddy CM. Social cognition and self-other distinctions in neuropsychiatry: insights from schizophrenia and tourette syndrome. Prog Neuropsychopharmacol Biol Psychiatry. (2018) 82:69–85. 10.1016/j.pnpbp.2017.11.026 29195921

[71] Lenzi D Trentini C Pantano P Macaluso E Iacoboni M Lenzi GL. Neural basis of maternal communication and emotional expression processing during infant preverbal stage. Cereb Cortex. (2009) 19:1124–33. 10.1093/cercor/bhn153 18787229

[72] Strathearn L Kim S Mothers' amygdala response to positive or negative infant affect is modulated by personal relevance. Front Neurosci. (2013) 7:176 10.3389/fnins.2013.00176 24115918 PMC3792358

[73] Kim S Fonagy P Allen J Strathearn L. Mothers' unresolved trauma blunts amygdala response to infant distress. Soc Neurosci. (2014) 9:352–63. 10.1080/17470919.2014.896287 24635646 PMC4260525

[74] Bradley MM Lang PJ. Measuring emotion: the self-assessment manikin and the semantic differential. J Behav Ther Exp Psychiatry. (1994) 25:49–59. 10.1016/0005-7916(94)90063-9 7962581

[75] Mclaren DG Ries ML Xu G Johnson SC. A generalized form of context-dependent psychophysiological interactions (gPPI): a comparison to standard approaches. Neuroimage. (2012) 61:1277–86. 10.1016/j.neuroimage.2012.03.068 22484411 PMC3376181

[76] Schilbach L Timmermans B Reddy V Costall A Bente G Schlicht T. Toward a second-person neuroscience. Behav Brain Sci. (2013) 36:393–414. 10.1017/S0140525X12000660 23883742

[77] Saxe R. Why and how to study theory of mind with fMRI. Brain Res. (2006) 1079:57–65. 10.1016/j.brainres.2006.01.001 16480695

[78] Swain JE Ho SS. Early postpartum resting-state functional connectivity for mothers receiving buprenorphine treatment for opioid use disorder: a pilot study. J Neuroendocrinol. (2019) 31:e12770. 10.1111/jne.12770 31287922 PMC7195812

[79] Maldjian JA Laurienti PJ Kraft RA Burdette JH. An automated method for neuroanatomic and cytoarchitectonic atlas-based interrogation of fMRI data sets. Neuroimage. (2003) 19:1233–9. 10.1016/S1053-8119(03)00169-1 12880848

[80] Tzourio-Mazoyer N Landeau B Papathanassiou D Crivello F Etard O Delcroix N. Automated anatomical labeling of activations in SPM using a macroscopic anatomical parcellation of the MNI MRI single-subject brain. Neuroimage. (2002) 15:273–89. 10.1006/nimg.2001.0978 11771995

[81] Cssp (2015). Strengthening Families™: A Protective Factors Framework. Available online at: http://www.cssp.org/reform/strengtheningfamilies (accessed August 16, 2015).

[82] Schuster M Mom Power Fidelity Scale (2013).

[83] Abidin R Parenting Stress index. Lutz, FL: Psychological Assessment Resources (1995).

[84] Reitman D Currier RO Stickle TR. A critical evaluation of the parenting stress index-short form (PSI-SF) in a head start population. J Clin Child Adol Psychol. (2002) 31:384–92. 10.1207/S15374424JCCP3103_10 12149976

[85] Barroso NE Hungerford GM Garcia D Graziano PA Bagner DM. Psychometric properties of the Parenting Stress Index-Short Form (PSI-SF) in a high-risk sample of mothers and their infants. Psychol Assess. (2016) 28:1331–5. 10.1037/pas0000257 26595220 PMC4877285

[86] Hayes AF Introduction to Mediation, Moderation, and Conditional Process Analysis, Second Edition: A Regression-Based Approach. New York, NY: Guilford Publications (2017).

[87] Kanwisher N Mcdermott J Chun MM. The fusiform face area: a module in human extrastriate cortex specialized for face perception. J Neurosci. (1997) 17:4302–11. 10.1523/JNEUROSCI.17-11-04302.1997 9151747 PMC6573547

[88] Eyal T Steffel M Epley N. Perspective mistaking: accurately understanding the mind of another requires getting perspective, not taking perspective. J Pers Soc Psychol. (2018) 114:547–71. 10.1037/pspa0000115 29620401

[89] Weinberg MS Grissom N Paul E Bhatnagar S Maier SF Spencer RL Inescapable but not escapable stress leads to increased struggling behavior and basolateral amygdala c-fos gene expression in response to subsequent novel stress challenge. Neuroscience. (2010) 170:138–48. 10.1016/j.neuroscience.2010.06.052 20600641 PMC2926237

[90] Leuner B Fredericks PJ Nealer C Albin-Brooks C. Chronic gestational stress leads to depressive-like behavior and compromises medial prefrontal cortex structure and function during the postpartum period. PLoS ONE. (2014) 9:e89912. 10.1371/journal.pone.0089912 24594708 PMC3940672

[91] Mcewen AM Burgess DTA Hanstock CC Seres P Khalili P Newman SC. Increased glutamate levels in the medial prefrontal cortex in patients with postpartum depression. Neuropsychopharmacology. (2012) 37:2428. 10.1038/npp.2012.101 22805604 PMC3442339

[92] Swain JE Tasgin E Mayes LC Feldman R Constable RT Leckman JF. Maternal brain response to own baby-cry is affected by cesarean section delivery. J Child Psychol Psychiatry. (2008) 49:1042–52. 10.1111/j.1469-7610.2008.01963.x 18771508 PMC3246837

[93] Ho SS Swain JE. Depression alters maternal extended amygdala response and functional connectivity during distress signals in attachment relationship. Behav Brain Res. (2017) 325:290–6. 10.1016/j.bbr.2017.02.045 28263829 PMC5446941

[94] Atzil S Hendler T Feldman R. Specifying the neurobiological basis of human attachment: brain, hormones, and behavior in synchronous and intrusive mothers. Neuropsychopharmacology. (2011) 36:2603–15. 10.1038/npp.2011.172 21881566 PMC3230500

[95] Barrett J Wonch KE Gonzalez A Ali N Steiner M Hall GB. Maternal affect and quality of parenting experiences are related to amygdala response to infant faces. Soc Neurosci. (2012) 7:252–68. 10.1080/17470919.2011.609907 21943083

[96] Young KD Siegle GJ Bodurka J Drevets WC. Amygdala activity during autobiographical memory recall in depressed and vulnerable individuals: association with symptom severity and autobiographical overgenerality. Am J Psychiatry. (2016) 173:78–89. 10.1176/appi.ajp.2015.15010119 26541813

[97] Brown S Martinez MJ Parsons LM. The neural basis of human dance. Cerebral Cortex. (2006) 16:1157–67. 10.1093/cercor/bhj057 16221923

[98] Bornstein MH Putnick DL Rigo P Esposito G Swain JE Suwalsky JTD Neurobiology of culturally common maternal responses to infant cry. Proc Natl Acad Sci USA. (2017) 114:E9465–E9473. 10.1073/pnas.1712022114 29078366 PMC5692572

[99] Kim P Rigo P Leckman JF Mayes LC Cole PM Feldman R. A prospective longitudinal study of perceived infant outcomes at 18-24 months: neural and psychological correlates of parental thoughts and actions assessed during the first month postpartum. Front Psychol. (2015) 6:1772. 10.3389/fpsyg.2015.01772 26635679 PMC4654106

[100] Azhari A Leck WQ Gabrieli G Bizzego A Rigo P Setoh P. Parenting stress undermines mother-child brain-to-brain synchrony: a hyperscanning study. Sci Rep. (2019) 9:11407. 10.1038/s41598-019-47810-4 31388049 PMC6684640

[101] Friston K Am i self-conscious? (or does self-organization entail self-consciousness?). Front Psychol. (2018) 9:579 10.3389/fpsyg.2018.00579 29740369 PMC5928749

[102] Hommel B Musseler J Aschersleben G Prinz W. The theory of event coding (TEC): a framework for perception and action planning. Behav Brain Sci. (2001) 24:849–78. 10.1017/S0140525X01000103 12239891

[103] He BJ Raichle ME. The fMRI signal, slow cortical potential and consciousness. Trends Cogn Sci. (2009) 13:302–9. 10.1016/j.tics.2009.04.004 19535283 PMC2855786

[104] Mashour GA. Cognitive unbinding: a neuroscientific paradigm of general anesthesia and related states of unconsciousness. Neurosci Biobehav Rev. (2013) 37:2751–9. 10.1016/j.neubiorev.2013.09.009 24076246 PMC3870022

[105] Bachmann T Hudetz AG. It is time to combine the two main traditions in the research on the neural correlates of consciousness: C = L x D. Front Psychol. (2014) 5:940. 10.3389/fpsyg.2014.00940 25202297 PMC4141455

[106] Barrett LF Simmons WK. Interoceptive predictions in the brain. Nat Rev Neurosci. (2015) 16:419–29. 10.1038/nrn3950 26016744 PMC4731102

[107] Barrett LF Satpute AB. Historical pitfalls and new directions in the neuroscience of emotion. Neurosci Lett. (2019) 693:9–18. 10.1016/j.neulet.2017.07.045 28756189 PMC5785564

[108] Hutchinson JB Barrett LF. The power of predictions: an emerging paradigm for psychological research. Curr Dir Psychol Sci. (2019) 28:280–91. 10.1177/0963721419831992 31749520 PMC6867616

[109] Campbell JO. Universal darwinism as a process of bayesian inference. Front Syst Neurosci. (2016) 10:49. 10.3389/fnsys.2016.00049 27375438 PMC4894882

[110] Silver D Hubert T Schrittwiesser J Antonoglou I Lai M Guez A Mastering chess and shogi by self-play with a general reinforcement learning algorithm. arXiv [Preprint]. (2017). arXiv:1712.01815.

[111] Silver D Schrittwieser J Simonyan K Antonoglou I Huang A Guez A. Mastering the game of go without human knowledge. Nature. (2017) 550:354–9. 10.1038/nature24270 29052630

[112] Arioli M Perani D Cappa S Proverbio AM Zani A Falini A. Affective and cooperative social interactions modulate effective connectivity within and between the mirror and mentalizing systems. Hum Brain Mapp. (2018) 39:1412–27. 10.1002/hbm.23930 29265483 PMC6866589

[113] Schacter D Addis DR Hassabis D Martin VC Spreng RN Szpunar KK. The future of memory: remembering, imagining, and the brain. Neuron. (2012) 76:677–94. 10.1016/j.neuron.2012.11.001 23177955 PMC3815616

