# Supplementary material for: Potential Neural Mediators of Mom Power Parenting Intervention Effects on Maternal Intersubjectivity and Stress Resilience
Source: Front Psychiatry. 2020 Dec 8;11:568824. doi: 10.3389/fpsyt.2020.568824 (PMC7752922; doi:10.3389/fpsyt.2020.568824)
Supplement: Supplementary file 1 [file Data_Sheet_1.DOCX]

***Supplementary Material***

Title: Potential Neural Mediators of Mom Power Parenting Intervention Effects on Maternal Intersubjectivity and Stress Resilience

Authors: S. Shaun Ho^1^, Maria Muzik^2^, Katherine L Rosenblum^2^, Diana Morelen^3^, Yoshio Nakamura^4^, James E. Swain^1^

**Brief Introduction of Mom Power Intervention**

The Mom Power (MP) curriculum has been described in detail previously ([Muzik et al., 2015a](#_ENREF_3);[Muzik et al., 2016](#_ENREF_5);[Rosenblum et al., 2018](#_ENREF_6)). Paralleling the Strengthening Families Protective Factors Framework ([CSSP, 2015](#_ENREF_2);[Muzik et al., 2015b](#_ENREF_4)), MP is an attachment-based 13-session psychotherapeutic intervention that aims to: 1) enhance sensitive and nurturing

parenting, 2) reduce caregivers’ mental health problems, and 3) promote parental reflective functioning in high-risk mothers. The program rests upon five core “pillars”:

1. Enhancing peer and other social supports
2. An attachment-based parenting curriculum
3. Stress regulation and self-care practice
4. Practice and guided support for parent-child interaction
5. Connecting to community resources, including professional care for wellbeing

Although session content varies week to week, the focus of the attachment-based parenting education is on promoting positive parenting and secure relationships between parent and child. MP encourages mothers to increase reflective capacity and gain insight through

supportive exposure to attachment-based parenting principles. While manualized, the curriculum is highly individualized, interactive and experiential, and delivered in a group format designed to create a welcoming, trust-building atmosphere. Parents learn how to function as a secure base and a safe haven for their children ([Bowlby, 1988](#_ENREF_1)); how to be empathic to their children’s emotional needs and experiences; how to repair a disruption in the relationship; how to co-regulate their children’s emotions; and how to create an atmosphere of warmth, joy and delight in which their children can learn and grow. In addition, parents explore what past experiences might impact their parenting and what current experiences may be affecting their children.

**Independent raters' perception on the images used in the fMRI task**

The images used in the fMRI task were rated by four female raters who were blind to the grouping of the participants, with the means and s.e. of the valence and arousal ratings plotted in Figure S1. Using ANOVA models, we found a significant main effect of emotions on valence rating (*F_(3,39)_* = 32.6, *p* < 0.001), with post hoc contrasts confirming valence ratings of Distress and Joy expressions differed from each other and all other conditions (all *p* < .001), and Neutral and Ambiguous faces were not different (*p* = .227). There was no main effect of child's identity, Own and Other child's faces were also not different in any condition (all *p* > .70). Similarly, we found a significant main effect of emotions on arousal rating (*F_(3,39)_* = 34.9, *p* < 0.001), with post hoc contrasts confirming that arousal ratings of Distress and Joy were not different from each other (*p* = .204) but were both different from Neutral and Ambiguous (*p* < .001), and Neutral and Ambiguous were different from each other (*p* = .007). There was no main effect of child's identity as Own and Other's child pictures were not different in any conditions (all *p* > .70). See **Supplemental Fig. S1**.

**MP Treatment Effects on PSI**

We found, in Study 2, that the PSI Total did not differ between the groups at pre-treatment (MP: *M* = 84.09, *s.e.* = 6.51; Control: *M* = 70.15, *s.e.* = 6.78, *F_(1,23)_* = 2.20, *MS_error_* = 551.55, *p* = 0.15). In accordance with a previously reported clinical trial showing that MP reduced parenting stress ([Rosenblum et al., 2017](#_ENREF_7)), in a GLM with repeated measurement, using MP vs. Control Group as between-subject factor and Time as within-subject factor and PSI at T1 and T2 as dependent variables, we found that a marginally significant Group x Time interaction (*F_(1, 21)_* = 4.071, *MS_error_* = 99.578, *p* = 0.057). Because we previously found that the stress level at baseline may interact with the treatment effects ([Muzik et al., 2015a](#_ENREF_3)), we specifically tested the MP effects on the PSI at T2 in an ANCOVA one-tailed test, controlling for the baseline PSI at T1 as a covariate. We found that the PSI at T2 was significantly lower in MP than Control (MP: *M* = 73.93, *s.e.* = 4.12; Control: *M* = 85.53, *s.e.* = 4.74, *F_(1, 23)_* = 3.221, *MS_error_* = 208.807, *p* = 0.044). See **Supplemental Fig. S2**.

**The Main Effects of Task Condition on Cue Period**

In CFMT, to ensure the participants’ wakefulness and readiness for the task, the participants were cued to press a button to indicate their readiness to perform tasks of Join, Observe, and React. It is of interest to examine the task main effects in both Study 1 and 2, and the group (MP vs. Control) by time interaction effect in Study 2, on the reaction time (RT), which is the duration between the onset of a cue for each condition and the time of button press.

In Study 1, we found a significant Task main effect on RT (*F_(2, 82)_* = 9.222, MS_error_ = 221701.622, *p* < 0.001), with the RT for Observe was significantly shorter than those for Join and React (**Supplementary Fig. S3a**). In Study 2, we found a significant Task main effect on RT as well (*F_(2, 44)_* = 6.543, MS_error_ = 263728.091, *p* = 0.003). There was no Time main effect (*p* = 0.997), Group main effect (*p* = 0.471), or Group by Time interaction effect (*p* = 0.960) (**Supplementary Fig. S3b**).

These results suggested that the participants may require less efforts in Observe than other task conditions in order for them to get ready for the tasks.

**Study 2 Results After Excluding Participants with Anti-depressant Medication**

One way to address the concern of the potential confounding of anti-depressant medication in Study 2 was to compare the original fMRI results with new results after excluding anti-depressant medicated participants. We found that the exclusion did not affect the results qualitatively. As depicted side-by-side in **Supplementary Fig. S4**, all original results of significant group main effects, which are re-presented in **Supplementary Fig. S4a**, remained significant (*p* < 0.05, denoted with *), or marginally significant (*p* < 0.10, denoted with #), in the results after exclusion (**Supplementary Fig. S4b**).

**Supplementary Fig. S1**


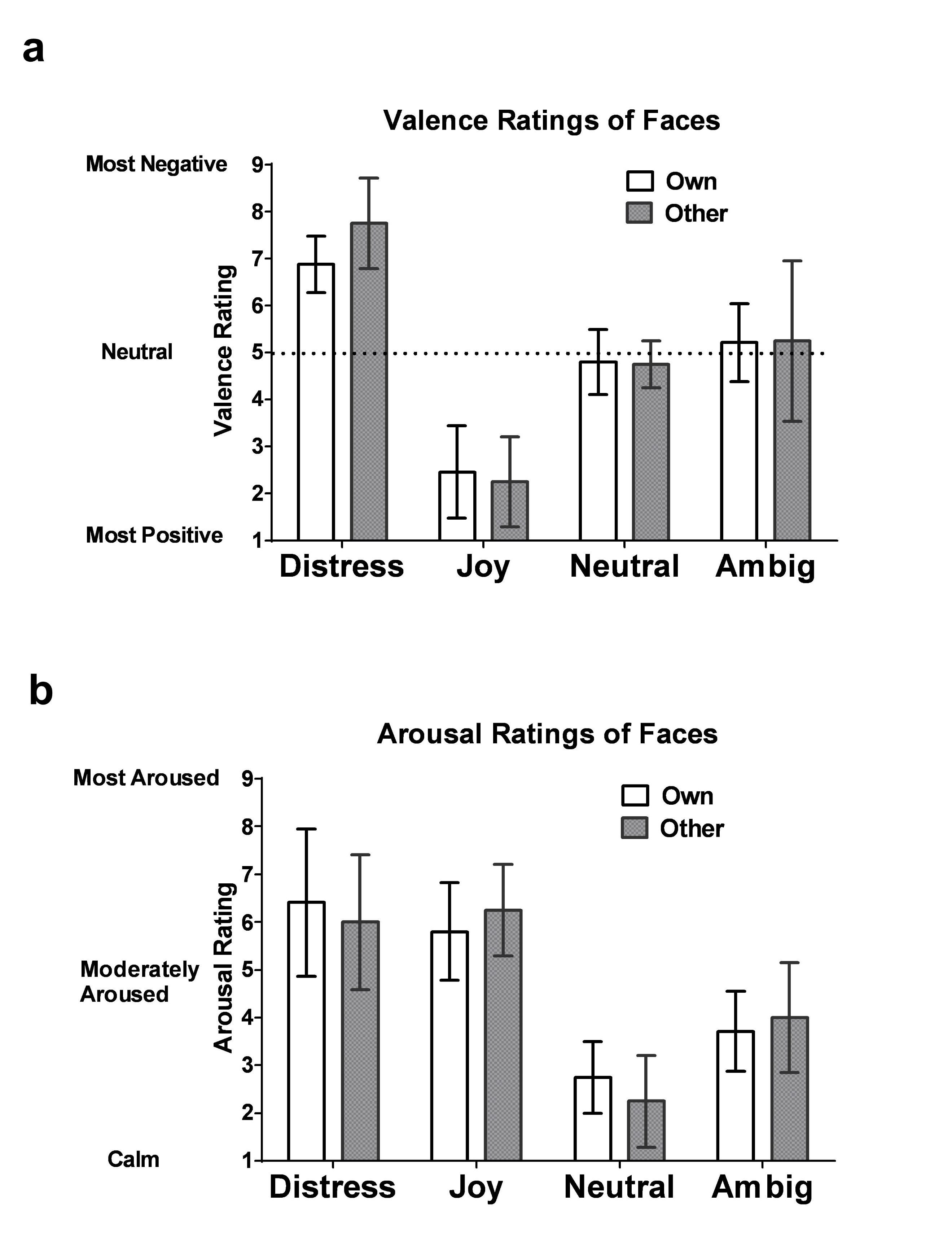


**Supplementary Fig. S1.** Bar charts of the independent raters' perception of valence (a) and arousal (b) on the images used in the fMRI task.

**Supplementary Fig. S2**


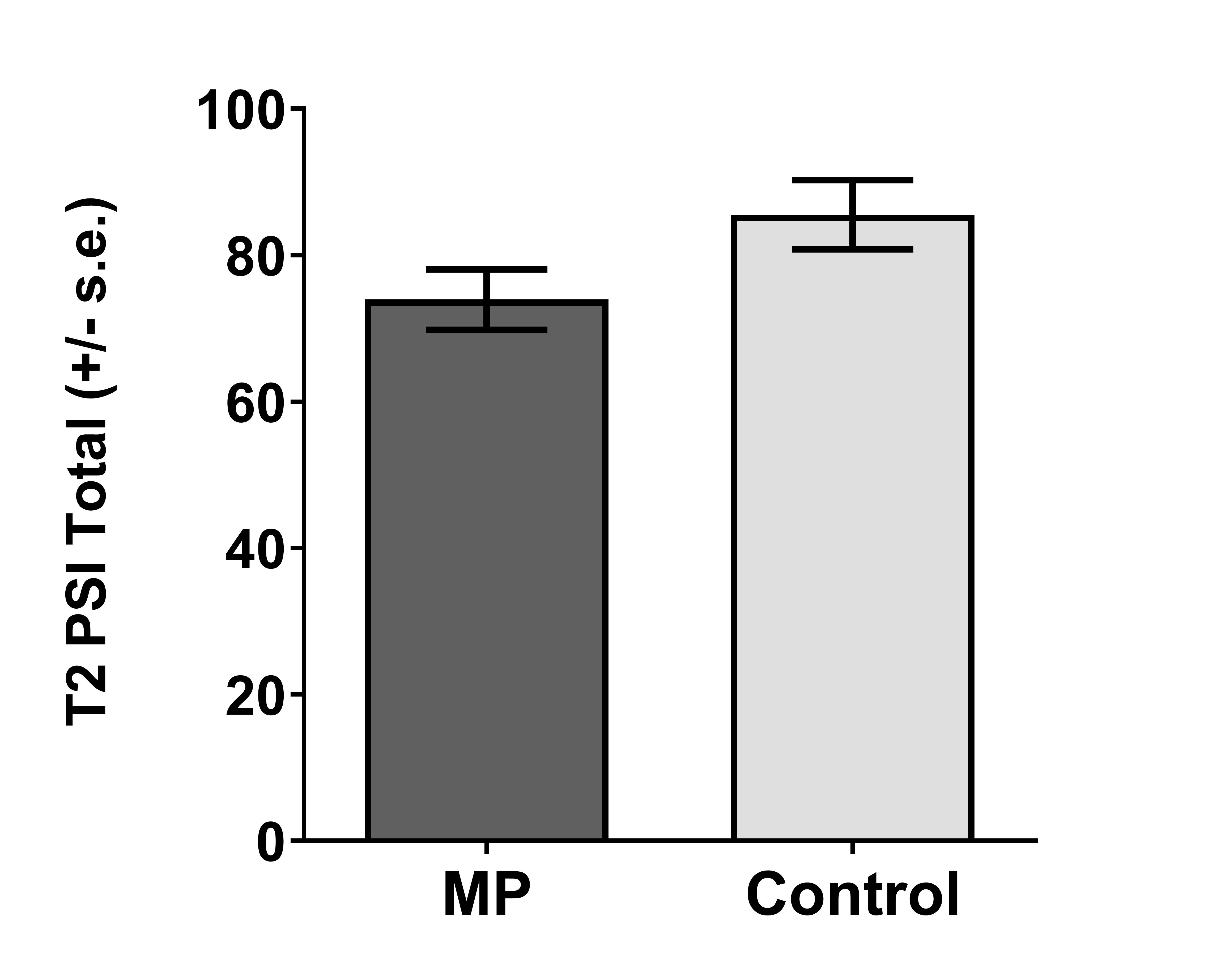


**Supplementary Fig. S2.** Parenting Stress Index (PSI Total) at post-treatment (T2), controlling for a covariate, pre-treatment PSI Total = 77.52.

**Supplementary Fig. S3**


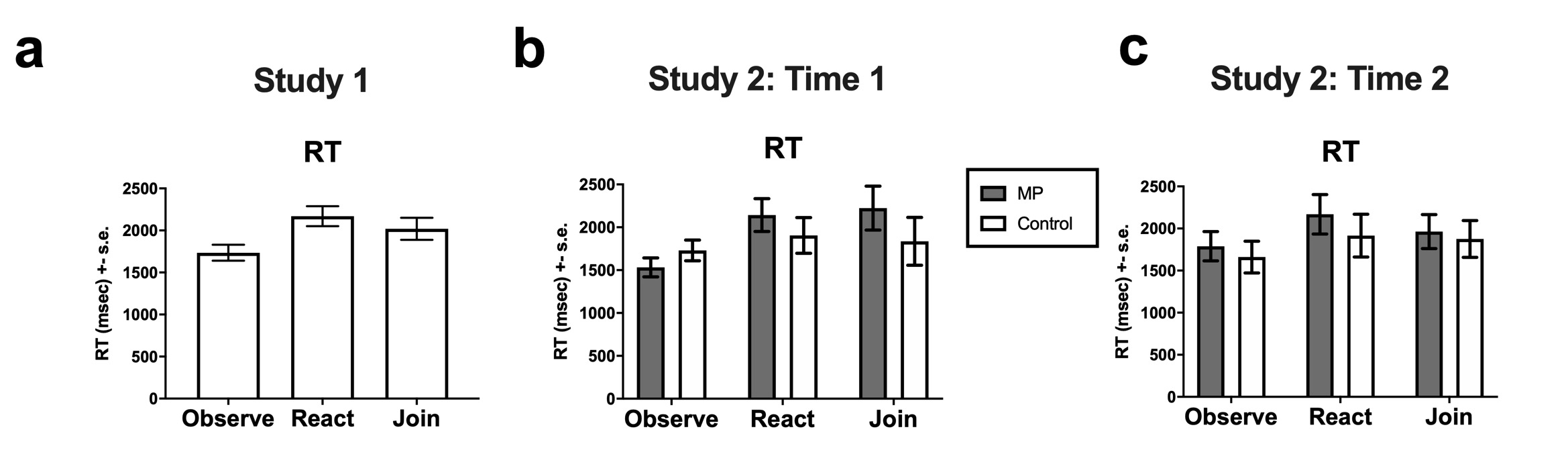


**Supplementary Fig. S3.** Bar charts of the averaged median RT in the Cue periods for each task condition in CFMT in Study 1 (a), Study 2 Time 1 (b), and Study 2 Time 2 (c).

**Supplementary Fig. S4**


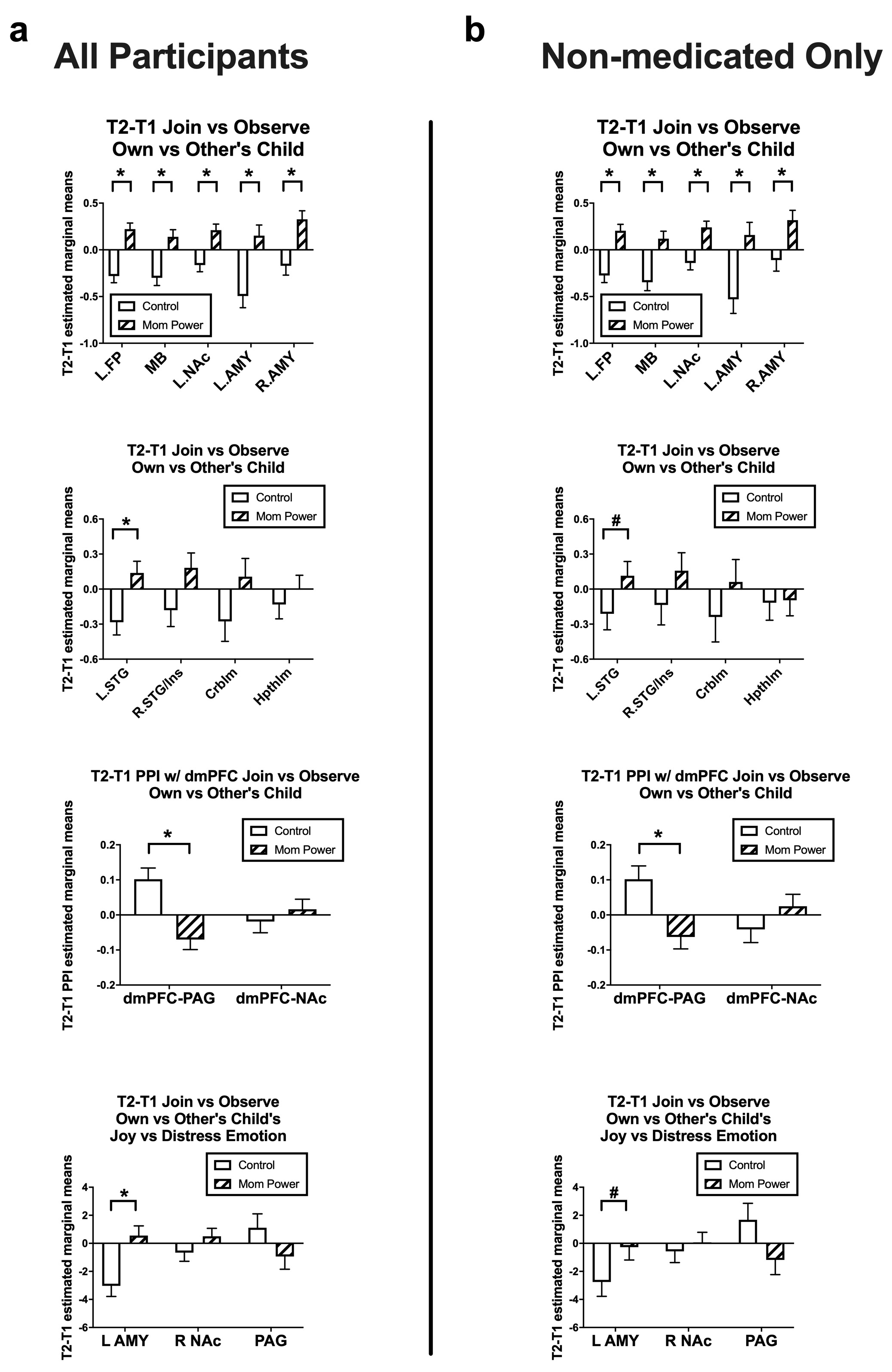


**Supplementary Fig. S4.** Side-by-side comparison between the original results (a) and new results after excluding medicated participants (b) in Study 2.

**Supplementary Fig. S5**


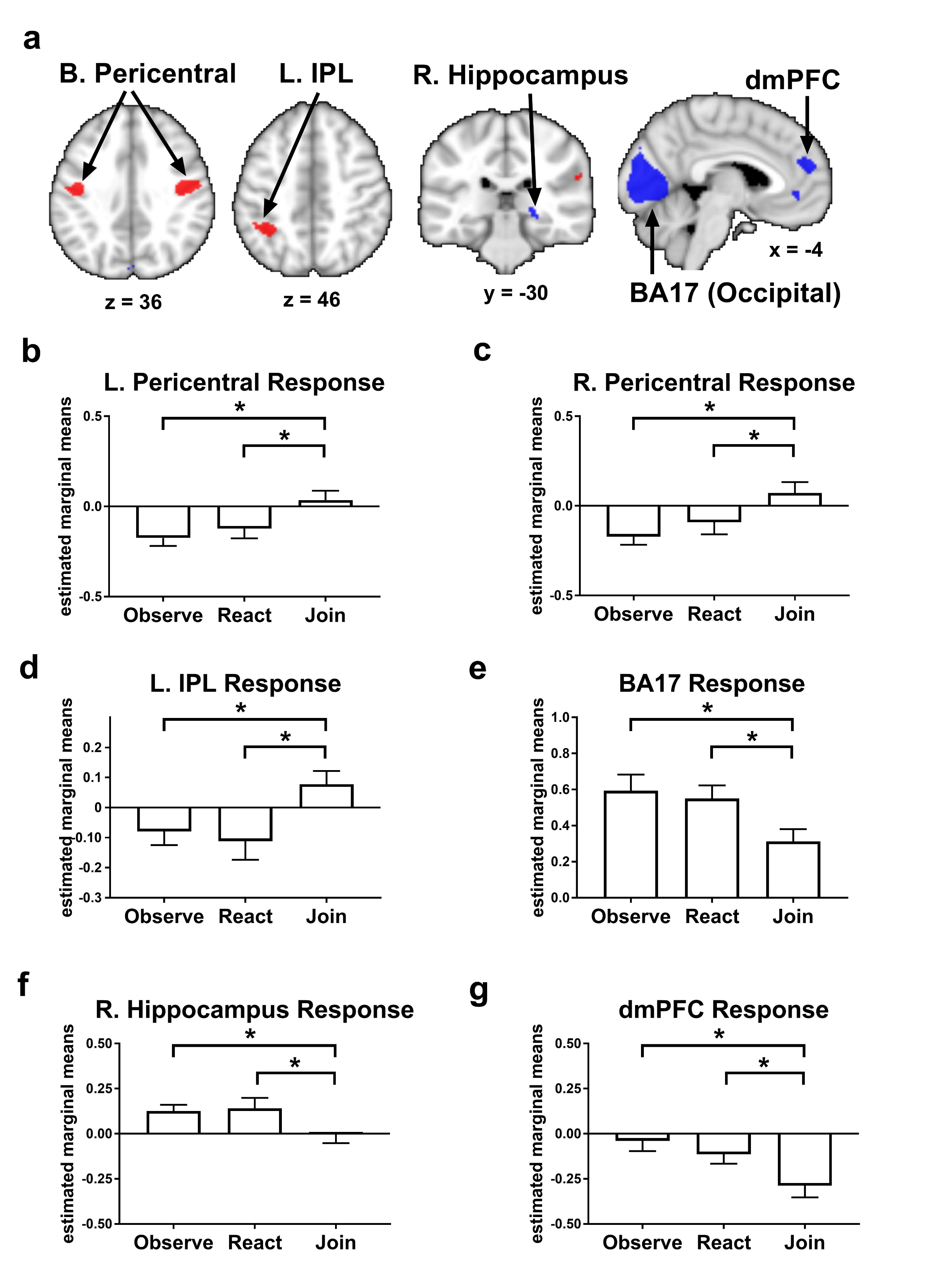


**Supplementary Fig. S5.** Bar charts of Study 1 mean neural responses in different regions in Observe, React, and. Join conditions.

**Supplementary Fig. S6**


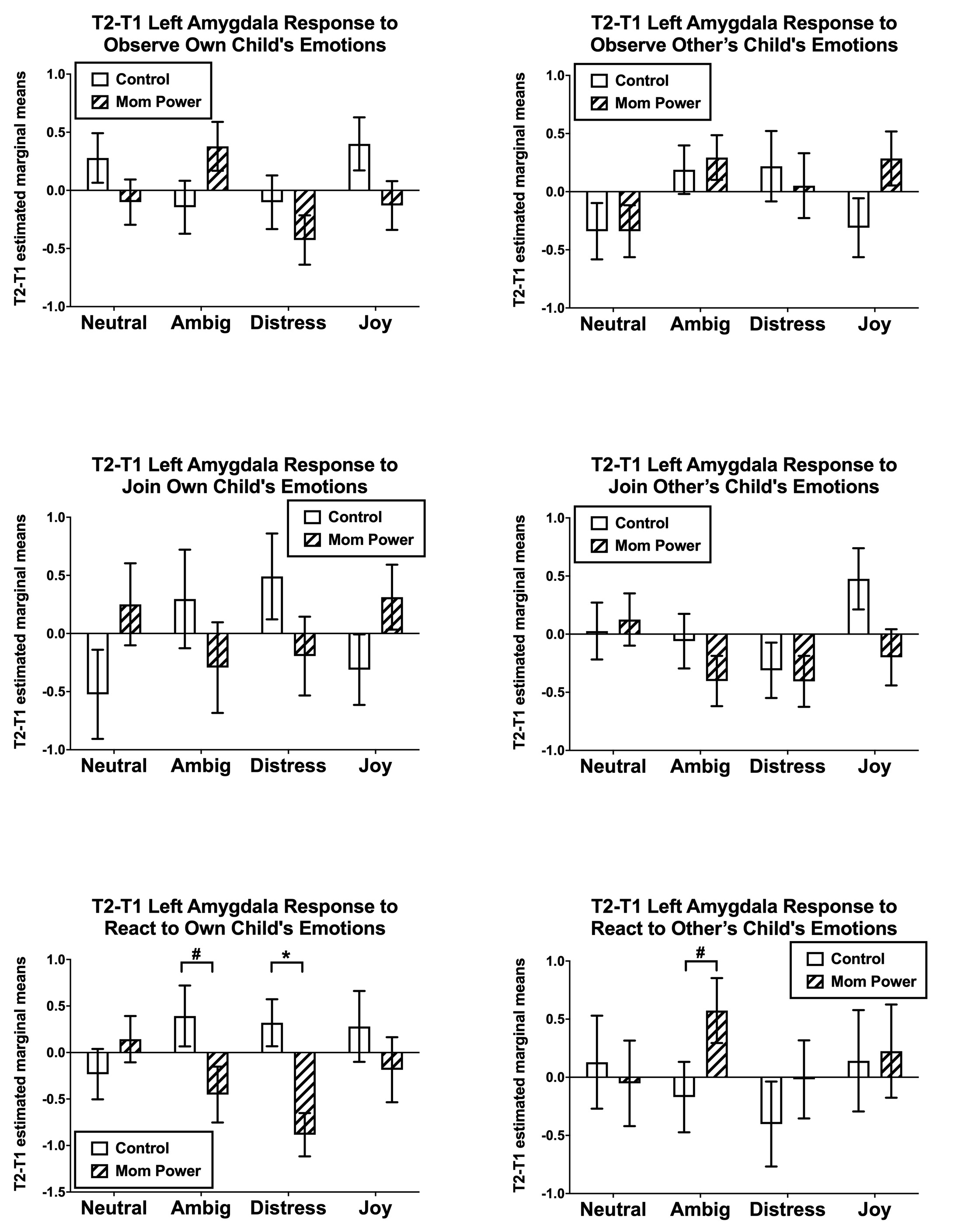


**Supplementary Fig. S6.** Bar charts of Study 2 T2-T1 differences in the left amygdala results in Observe, Join, React Own and Other’s child’s neutral, ambiguous, distressed, and joyful emotions, presented separately.

**Supplementary Fig. S7**


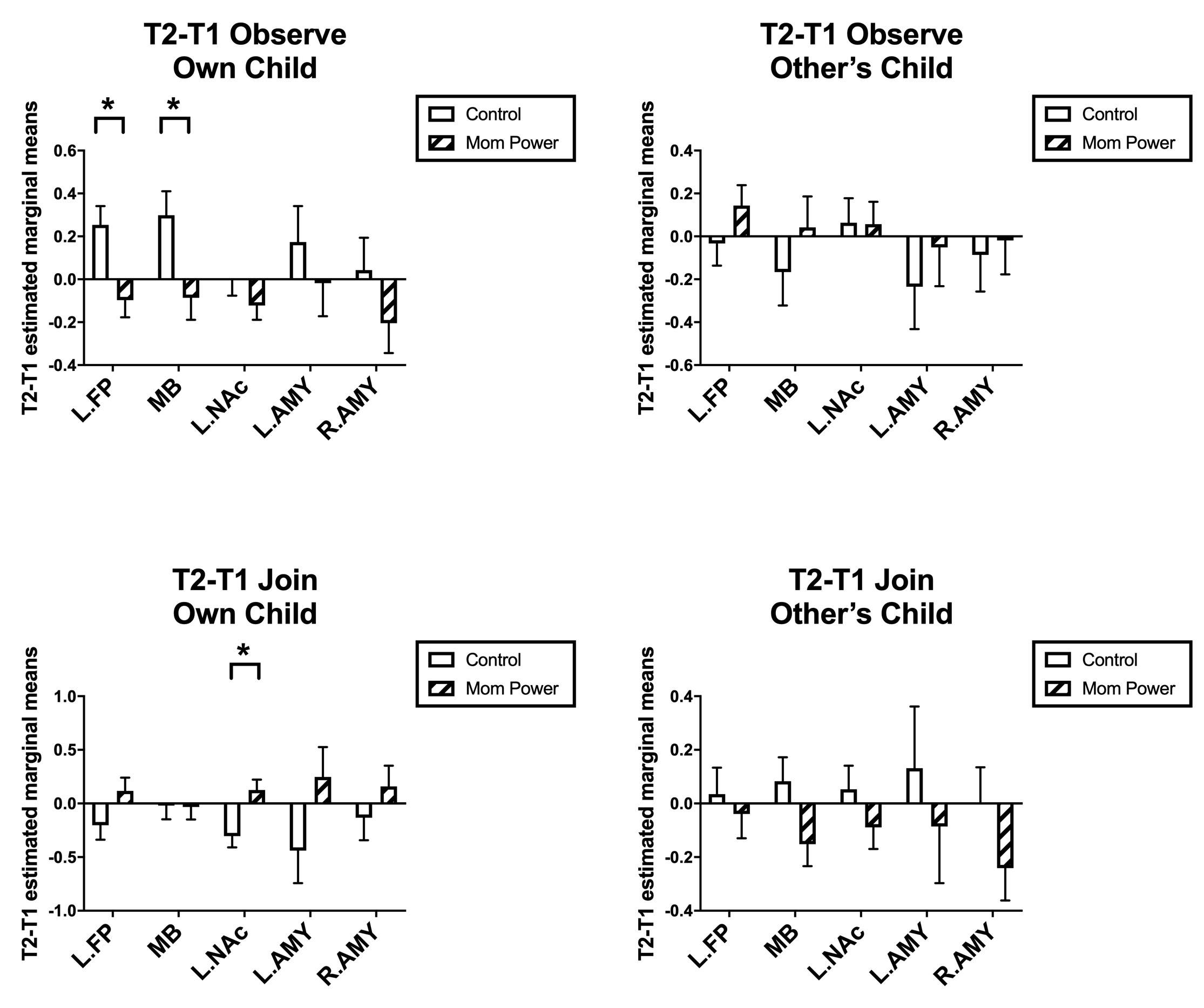


**Supplementary Fig. S7.** Bar charts of Study 2 T2-T1 differences in the left frontoparietal (L. FP), midbrain (MB), left nucleus accumbens (L. NAc) , left amgydala (L. AMY), and right amygdala (R. AMY) in the Observe and Join Own and Other’s Child’s emotions (all emotions combined), presented separately.

**Supplementary Fig. S8**


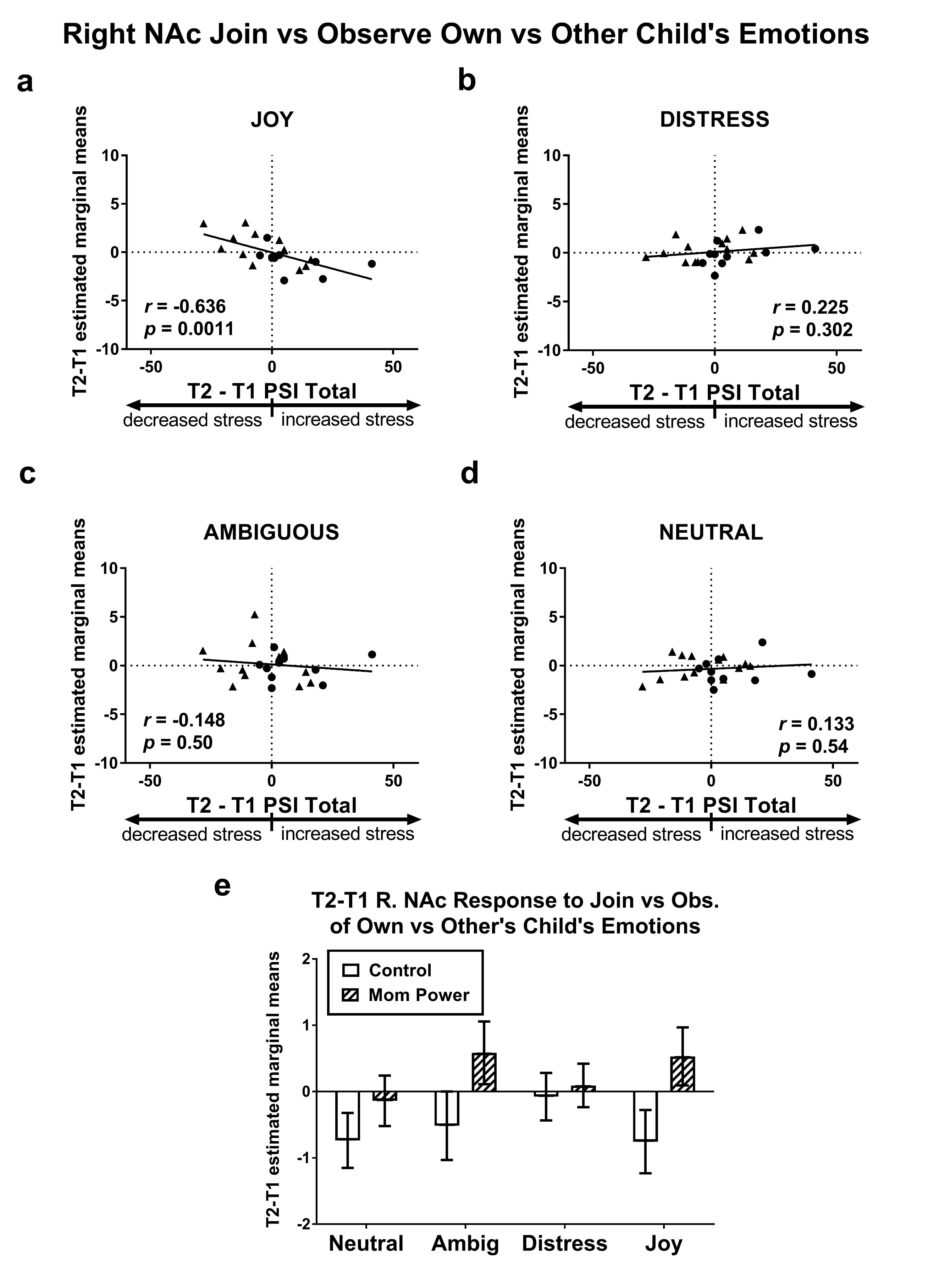


**Supplementary Fig. S8.** Scatter plots of Study 2 T2-T1 changes in PSI (x-axis) and T2-T1 differential responses in the right NAc (y-axis) in the contrasts of MMR(joy) (a), MMR(dist) (b), MMR(amb) (c), and MMR(neu) (d). Bar charts of T2-T1 right NAc MMR(all) responses (e).

**Supplementary Fig. S9**


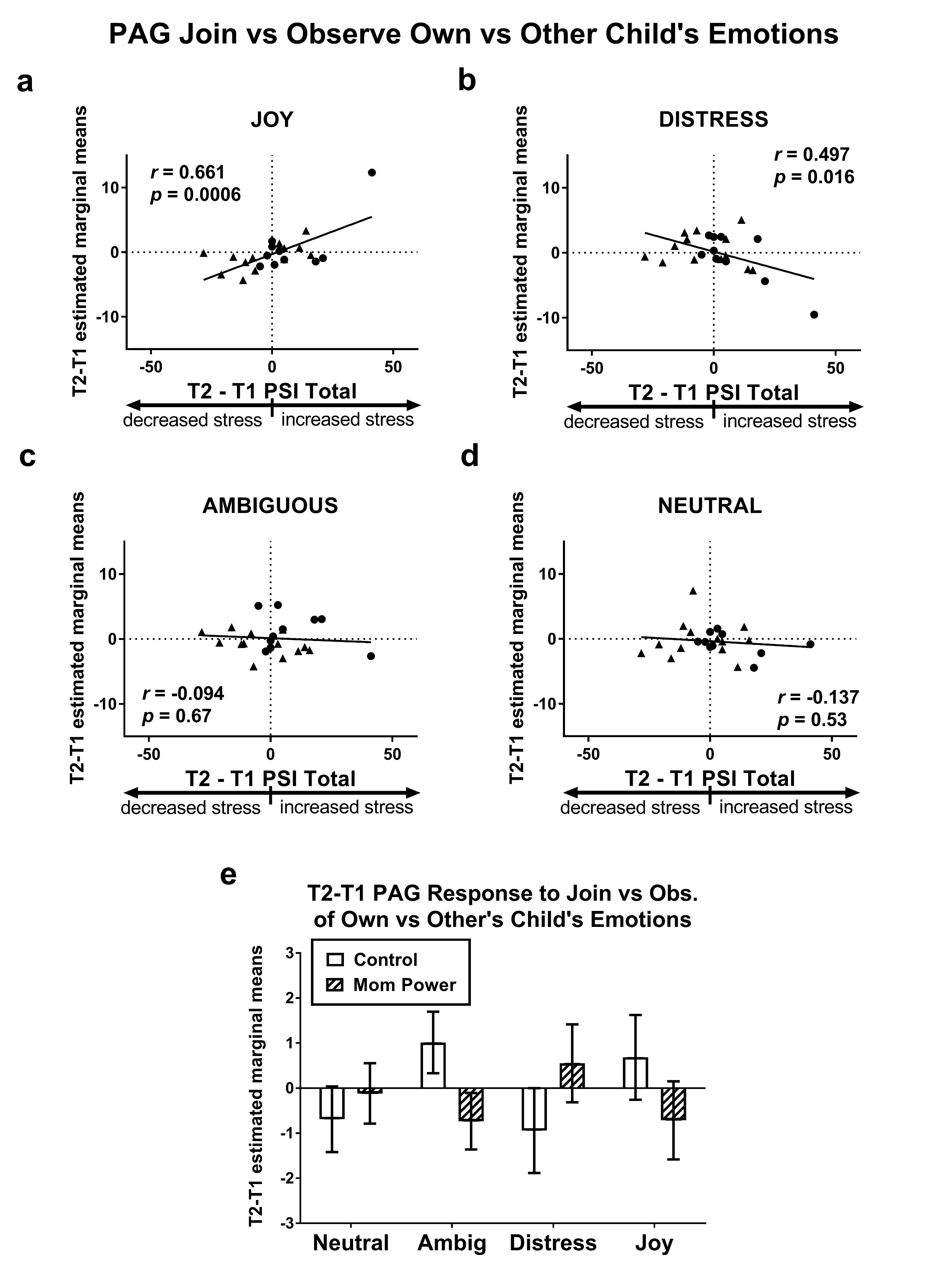


**Supplementary Fig. S9.** Scatter plots of Study 2 T2-T1 changes in PSI (x-axis) and T2-T1 differential responses in the PAG (y-axis) in the contrasts of MMR(joy) (a), MMR(dis) (b), MMR(amb) (c), and MMR(neu) (d). Bar charts of T2-T1 PAG MMR(all) responses (e).

**Supplementary Fig. S10**


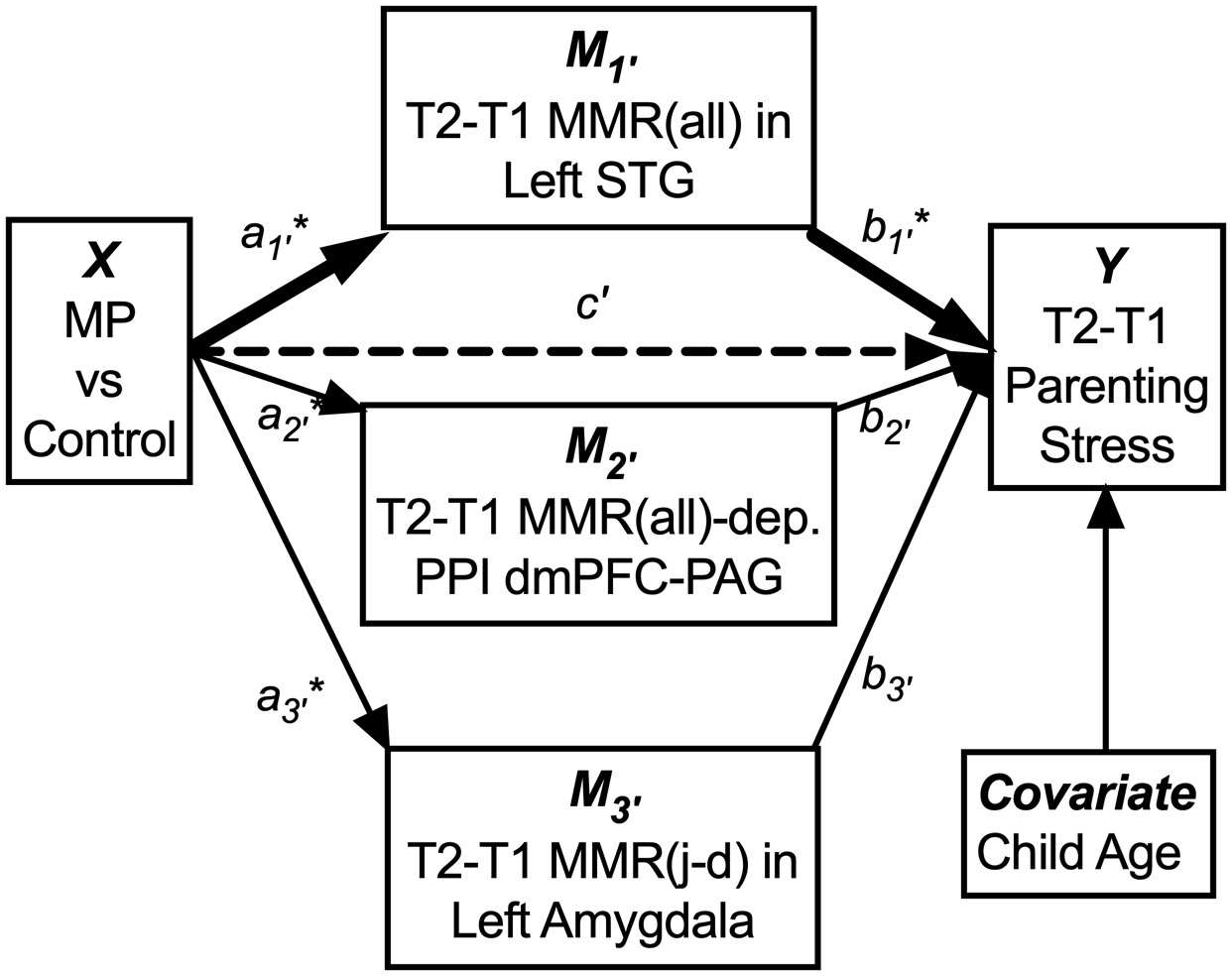


**Supplementary Fig. S10.** The three-mediator (M_1’_, M_2’_, and M_3’_) model showed that the relative indirect effect of M_1’_ was significant. The age of Own Child was used as a covariate. See Supplementary Table S3 for the statistical results of the three-mediator model.

| **Supplementary Table S1**  **Own vs. Other Child Effects** | | | | | | | | | |
| --- | --- | --- | --- | --- | --- | --- | --- | --- | --- |
|  |  | MNI Coordinates | | | | No. of Voxels | | |  |
| Brain Region | Side | X | Y | Z | |  |  |  | Z score |
|  |  |  |  | |  | |  |  | |
| **Own > Other Child Main Effect*** | | | | | | | | | |
| Occipital lobe | L | -22 | -94 | | 4 | | 848 | 4.71 | |
|  | R | 30 | -88 | | -2 | | 1089 | 4.30 | |
|  |  |  |  | |  | |  |  | |
| Precuneus | R/L | 10 | -66 | | 36 | | 1468 | 4.42 | |
|  |  |  |  | |  | |  |  | |
| FIO/IFG | L | -28 | 22 | | -20 | | 383 | 4.14 | |
|  | R | 40 | 40 | | -12 | | 242 | 3.91 | |
|  |  |  |  | |  | |  |  | |
| Angular gyrus | R | 34 | -70 | | 42 | | 405 | 4.12 | |
|  |  |  |  | |  | |  |  | |
| **Observe Own > Other Child (simple main effect)*** | | | | | | | | | |
| Fusiform face area (FFA) | R | 34 | -82 | | -4 | | 7503 | 5.35 | |
|  | L | -34 | -80 | | -6 | | 2546 | 4.96 | |
|  |  |  |  | |  | |  |  | |
| MFG/IFG | R | 42 | 34 | | 14 | | 522 | 4.45 | |
|  |  |  |  | |  | |  |  | |
| STG/FIO/Temporal pole | L | -34 | 12 | | -34 | | 1984 | 4.14 | |
|  | R | 52 | 10 | | -24 | | 1616 | 4.13 | |
| (including hippocampus, posterior) | L | -28 | -30 | | -10 | | 88 | 3.56 | |
| (including hippocampus, anterior) | L | -34 | -12 | | -16 | | 95 | 3.40 | |
|  |  |  |  | |  | |  |  | |
| SMA | R | 10 | 10 | | 56 | | 286 | 3.74 | |
|  |  |  |  | |  | |  |  | |
| **React to Own > Other Child (simple main effect)**** | | | | | | | | | |
| Subcortical regions | L | -24 | -24 | | -2 | | 1286 | 3.90 | |
| (Thalamus) |  |  |  | |  | | (142) |  | |
| (Thalamus) | R | 26 | -22 | | -4 | | (116) | 3.89 | |
| (Midbrain) | R/L | 4 | -24 | | -10 | | (420) | 3.74 | |
| (Lentiform nucleus) | L | -12 | 4 | | -4 | | (122) | 3.69 | |
|  | R | 18 | -8 | | -6 | | (70) | 3.63 | |
| (Hippocampus, posterior) | R | 26 | -24 | | -8 | | (54) | 3.67 | |
| (Hypothalamus) | L | -8 | -4 | | -4 | | (5) | 2.85 | |
|  |  |  |  | |  | |  |  | |
| **Join Own > Other Child (simple main effect)** | | | | | | | | | |
| None |  |  |  | |  | |  |  | |
|  |  |  |  | |  | |  |  | |
|  | | | | | | | | | |
| * Whole brain corrected at false-discovery rate (FDR) = 0.05 | | | | | | | | | |
| ** Cluster-level whole brain corrected (FWE<0.05), at voxel-wise *p* = 0.005 unc. | | | | | | | | | |
|  |  |  |  | |  | |  |  | |

| **Supplementary Table S2**  **Planned Tests for Task x Child Interaction Effects** | | | | | | | | | |
| --- | --- | --- | --- | --- | --- | --- | --- | --- | --- |
|  |  | MNI Coordinates | | | | No. of Voxels | | |  |
| Brain Region | Side | X | Y | Z | |  |  |  | Z score |
|  |  |  |  | |  | |  |  | |
| **Join > Observe [Own > Other Child]** | | | | | | | | | |
| None |  |  |  | |  | |  |  | |
|  |  |  |  | |  | |  |  | |
| **Observe > Join [Own > Other Child]**** | | | | | | | | | |
| Precuneus | R | 18 | -34 | | 50 | | 951 | 3.59 | |
|  | L | 0 | -52 | | 50 | | 661 | 3.24 | |
|  |  |  |  | |  | |  |  | |
| Fusiform | R | 30 | -58 | | -16 | | 726 | 3.56 | |
|  |  |  |  | |  | |  |  | |
| **React > Observe [Own > Other Child]** | | | | | | | | | |
| None |  |  |  | |  | |  |  | |
|  |  |  |  | |  | |  |  | |
| **Observe > React [Own > Other Child]** | | | | | | | | | |
| None |  |  |  | |  | |  |  | |
|  |  |  |  | |  | |  |  | |
| **Join > React [Own > Other Child]** | | | | | | | | | |
| None |  |  |  | |  | |  |  | |
|  |  |  |  | |  | |  |  | |
| **React > Join [Own > Other Child]**** | | | | | | | | | |
| Subcortical regions | L | -4 | -12 | | -4 | | 636 | 4.14 | |
| (Midbrain) |  |  |  | |  | | (207) |  | |
| (Extended amygdala/lentiform nucleus) | L | -12 | 6 | | -2 | | (110) | 3.53 | |
|  | | | | | | | | | |
| ** Cluster-level whole brain corrected (FWE<0.05), at voxel-wise *p* = 0.005 unc. | | | | | | | | | |
|  |  |  |  | |  | |  |  | |

**Supplementary Table S3**: Summary of the three-mediator model

| One  Model | Path-*a_i’_* | | | Path-*b_i’_* | | | Path-*c’* | | | Relative Indirect Effect  (Path-*a’_i_b’_i_*) | | | |
| --- | --- | --- | --- | --- | --- | --- | --- | --- | --- | --- | --- | --- | --- |
|  | *Coef.* | *s.e.* | *p* | *Coef.* | *s.e.* | *p* | *Coef.* | *s.e.* | *p* | *effect* | *s.e.* | LLCI | ULCI |
| *M_1’_* | 0.431 | 0.157 | **0.013** | -15.933 | 6.869 | **0.033** | 7.296 | 6.136 | 0.251 | **-6.861*** | 4.123 | -17.527 | -0.886 |
| *M_2’_* | -0.171 | 0.046 | **0.0013** | 39.808 | 28.709 | 0.184 |  |  |  | -6.97 | 6.202 | -24.532 | 1.442 |
| *M_3’_* | 3.452 | 1.063 | **0.004** | -1.784 | 1.266 | 0.177 |  |  |  | -6.157 | 5.831 | -17.222 | 5.527 |
| **Notes:**  *M_1’_*: T2-T1 MMR(all) in the left STG;  *M_2’_*: T2-T1 MMR(all)-dependent PPI between dmPFC-PAG  *M_3’_*: T2-T1 MMR(j-d) in the left amygdala  *: 95% confidence interval did not cover zero  LLCI/ULCI: Lower/upper limit of 95% confidence interval | | | | | | | | | | | | | |

**References**

Bowlby, J. (1988). *A Secure Base: Parent-Child Attachment and Healthy Human Development.* London: Routledge.

Cssp (2015). *Strengthening Families™: A Protective Factors Framework* [Online]. Available: <http://www.cssp.org/reform/strengtheningfamilies> [Accessed August 16, 2015].

Muzik, M., Rosenblum, K.L., Alfafara, E.A., Schuster, M.M., Miller, N.M., Waddell, R.M., and Kohler, E.S. (2015a). Mom Power: preliminary outcomes of a group intervention to improve mental health and parenting among high-risk mothers. *Arch Womens Ment Health*.

Muzik, M., Rosenblum, K.L., Alfafara, E.A., Schuster, M.M., Miller, N.M., Waddell, R.M., and Kohler, E.S. (2015b). Mom Power: preliminary outcomes of a group intervention to improve mental health and parenting among high-risk mothers. *Archives of Women's Mental Health*.

Muzik, M., Rosenblum, K.L., Schuster, M.M., Kohler, E.S., Alfafara, E.A., and Miller, N.M. (2016). A mental health and parenting intervention for adolescent and young adult mothers and their infants. *Journal of Depression and Anxiety* 5**,** 233-239.

Rosenblum, K.L., Lawler, J., Alfafara, E., Miller, N., Schuster, M., and Muzik, M. (2018). Improving Maternal Representations in High-Risk Mothers: A Randomized, Controlled Trial of the Mom Power Parenting Intervention. *Child Psychiatry & Human Development* 49**,** 372-384.

Rosenblum, K.L., Muzik, M., Morelen, D.M., Alfafara, E.A., Miller, N.M., Waddell, R.M., Schuster, M.M., and Ribaudo, J. (2017). A community-based randomized controlled trial of Mom Power parenting intervention for mothers with interpersonal trauma histories and their young children. *Arch Womens Ment Health*.
